# OPTN is a host intrinsic restriction factor against neuroinvasive HSV-1 infection

**DOI:** 10.1038/s41467-021-25642-z

**Published:** 2021-09-13

**Authors:** Joshua Ames, Tejabhiram Yadavalli, Rahul Suryawanshi, James Hopkins, Alexander Agelidis, Chandrashekhar Patil, Brian Fredericks, Henry Tseng, Tibor Valyi-Nagy, Deepak Shukla

**Affiliations:** 1 Department of Microbiology and Immunology, University of Illinois at Chicago, College of Medicine, Chicago, IL USA; 2 Department of Ophthalmology and Visual Sciences, University of Illinois at Chicago, College of Medicine, Chicago, IL USA; 3 Department of Pathology, University of Illinois at Chicago, College of Medicine, Chicago, IL USA; 4 Duke Eye Center and Department of Ophthalmology, Duke University Medical Center, Durham, NC USA

**Keywords:** Chaperone-mediated autophagy, Necroptosis, Neuroimmunology, Viral host response, Viral infection

## Abstract

Fast-replicating neurotropic herpesviruses exemplified by herpes simplex virus-1 (HSV-1) naturally infect the central nervous system (CNS). However, most individuals intrinsically suppress the virus during a primary infection and preclude it from significantly damaging the CNS. Optineurin (OPTN) is a conserved autophagy receptor with little understanding of its role in neurotropic viral infections. We show that OPTN selectively targets HSV-1 tegument protein, VP16, and the fusion glycoprotein, gB, to degradation by autophagy. OPTN-deficient mice challenged with HSV-1 show significant cognitive decline and susceptibility to lethal CNS infection. OPTN deficiency unveils severe consequences for recruitment of adaptive immunity and suppression of neuronal necroptosis. Ocular HSV-1 infection is lethal without OPTN and is rescued using a necroptosis inhibitor. These results place OPTN at the crux of neuronal survival from potentially lethal CNS viral infections.

## Abstract (web summary)

During herpesvirus infection, most individuals intrinsically suppress a primary infection and therewith preclude potential damage or neurodegeneration of the CNS. Here, Ames et al. show that Optineurin (OPTN), a conserved autophagy receptor, restricts HSV-1 spread, degrades viral VP16 through autophagy and is neuroprotective against HSV infection in vivo.

## Introduction

Herpesviruses cause widespread infections that can lead to encephalitis, and evidence suggests herpesvirus infections of the central nervous system (CNS) contribute to the complex etiology of neurodegenerative disorders of the brain and eye^1–5^. Immunocompetent individuals are protected, but defects in innate or adaptive immunity may lead to death of infected neurons^6,7^. We highlight Optineurin (OPTN)-dependent selective autophagy as a critical intrinsic immune barrier against neurotropic viruses. Selective autophagy is required for specific degradation of damaging cellular occupants and is implicated in protecting the CNS from neurodegenerative diseases^8–10^.

Intracellular cargoes are ubiquitinated for binding to autophagy receptors, and the affinity of the receptors are enhanced through phosphorylation by TANK binding kinase 1 (TBK1)^11–13^. OPTN is an autophagy receptor which facilitates degradation of mitochondria and bacteria^11,14,15^. Mutations in OPTN have been identified, implicating it in human diseases including glaucoma, amyotrophic lateral sclerosis (ALS), Huntington’s disease, and Alzheimer’s disease (AD)^5,16–18^. Evidence supports the protective role of autophagy against protein aggregation in neurons, and it is suggested that it is an innate immune defense mechanism of the CNS^19,20^.

Selective autophagy can regulate signaling pathways^8,21–23^. OPTN negatively regulates necroptosis by degrading receptor interacting serine/threonine kinase 1 (RIPK1), protecting against neurodegeneration^24^. Autophagy has been associated with protection from interferon gamma (IFNγ) and tumor necrosis factor-alpha (TNF-α) mediated cell death^25^. This potential for selective autophagy to regulate inflammation and cell death may influence the outcome of CNS infections. Additionally, herpesviruses express factors to circumvent autophagy. HSV-1 encodes a virulence factor, γ_1_34.5 to inhibit autophagy^26^. Our study uses *Optn*−/− in vitro and in vivo models to examine the role of OPTN in the context of CNS immunity.

In this work we show that the host protein, OPTN, restricts HSV-1 infection in vitro and in vivo. OPTN deficiency leads to increased levels of the essential HSV-1 proteins, VP16, and gB. Furthermore, OPTN deficiency leads to diminished host immune responses to ocular HSV-1 infection, increased susceptibility to herpes encephalitis, and long-term loss of cognition.

## Results

### OPTN restrict spread of HSV-1

**Fig. 1:**
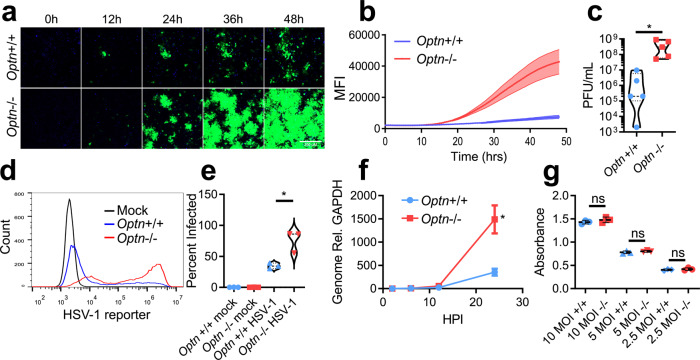
OPTN restricts spread of HSV-1. **a** Representative time-lapse imaging of *Optn*+/+ and −/− cells infected with GFP-HSV-1 17-strain at 0.1 MOI for 48 h. Time interval was 30 min. Scale bar is 250 µm. **b** Quantification of average time-lapse fluorescent intensity over time for three replicates. **c** Violin plot of *Optn*+/+ and −/− cells infected with HSV-1 at 0.1 MOI and assayed for plaques 24 hpi. *n* = 5 independent replicates per group. *P* = 0.0446. **d**, **e** (left) Histogram of *Optn*+/+ and −/− cells infected with GFP- HSV-1 at 0.1 MOI 24 hpi measured by flow cytometry, (right) violin plot of percentage of cells infected with GFP-HSV-1. *n* = 3 independent replicates per group. *P* = 0.0174. **f** Genomes present in *Optn*+/+ and −/− cells infected with GFP- HSV-1 at 0.1 MOI. *n* = 3 independent replicates per group. *P* = 0.0220. **g** Colorimetric readout of replication deficient and β-galactosidase expressing HSV-1 infected *Optn*+/+ and −/− cells at multiple MOIs. *n* = 3 independent replicates per group. *P* = 0.3083 (10 MOI), *P* = 0.3575 (5 MOI), *P* = 0.4945 (2.5 MOI). Data are presented as mean values ± SEM (**b**, **f**). Two-tailed Student’s *t* test was performed for statistical analysis (*α* = 0.05). ^∗^*p* < 0.05; ns not significant. Source data are provided as a Source Data file.

*Optn*−/− HeLa cells support rapid spread and replication of HSV-1. Time-lapse microscopy of GFP expressing HSV-1 revealed spread of infection to a larger population of cells in *Optn*−/− relative to *Optn*+/+ cells (Fig. 1a, b). By 24 hpi the production of infectious virus and viral genome replication was increased *Optn*−/− cells by ~100-fold and 5-fold respectively because of the enhanced viral spread (Fig. 1c, f). The percentage of infected cells was higher in the *Optn*−/− cell line 24 hpi (Fig. 1d, e) but entry of virus into cells was similar for both *Optn*−/− and *Optn*+/+ (Fig. 1g), thus ruling out different entry rates as an explanation for the increased viral spread. To further support the role of OPTN, mouse embryonic fibroblast (MEF) cells were derived from *Optn*+/+ or −/− mouse strains. *Optn*−/− MEF cells expressed significantly more late gene promoter signal when infected with HSV-1 KOS-strain (Supplementary Fig. 2A–C). When infected with HSV-1 17-strain, *Optn*−/− MEF cells showed significantly more widespread infection (Supplementary Fig. 2D–E). These results were further supported by infection of human corneal epithelial (HCE) cells and Lund human mesencephalic (LUHMES) cells that had been transfected with an siRNA targeting OPTN expression (Supplementary Fig. 1). Using multiple HSV-1 strains and pseudorabies virus (PRV), all expressing fluorescent reporters, we observed that OPTN generally restricts the spread of alphaherpesviruses in vitro. However, the differentiated neurons derived from LUHMES showed strain and virus specific differences in reporter signal. HCE, but not LUHMES, cells showed significantly more HSV-1 17 strain infection when OPTN was knocked down (Supplementary Fig. 2F–J). When infected with a dual reporter HSV-1 KOS strain, the reporter for HSV-1 late gene expression was significantly more expressed in both HCE and LUHMES cells where OPTN was knocked down, but only the LUHMES cells showed significantly more early gene reporter expression (Supplementary Fig. 2K–P). When infected with PRV expressing GFP under a CMV promoter, and RFP fused to the capsid protein, only capsid levels were significantly higher in OPTN knockdown HCE cells (Supplementary Fig. 2Q–V). Overall OPTN restricts infection, and this trend was observed for multiple HSV-1 strains and PRV in diverse cell types.

### OPTN is required for selective degradation of essential HSV-1 proteins

**Fig. 2:**
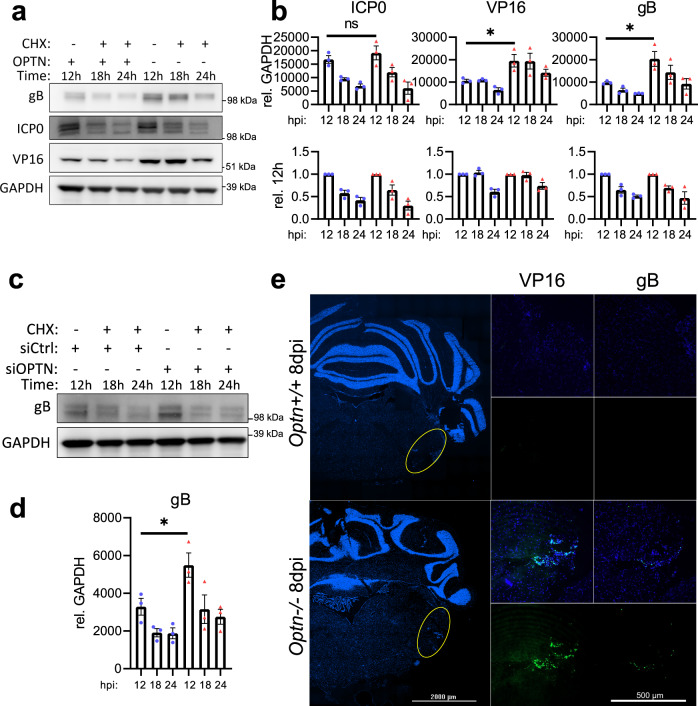
OPTN suppresses expression of essential HSV-1 proteins VP16 and gB, but not ICP0. *Optn*+/+ and −/− HeLa cells or siOPTN and siCtrl transfected HCE cells were infected at 1 MOI with HSV-1 17-strain for 12 h before CHX addition to block protein synthesis, then cells were sampled at 12 h, 18 h, and 24 h after infection. **a** Shown for HeLa cells are immunoblots against the HSV-1 proteins gB, ICP0, VP16, and the internal reference GAPDH, **b** with band quantification relative to GAPDH or relative to initial normalized protein levels (*n* = 3 replicates). *P* = 0.4591 (ICP0), *P* = 0.0368 (VP16), *P* = 0.0347 (gB). **c** Shown for HCE cells are immunoblots against the HSV-1 protein gB and the internal reference GAPDH, **d** with band quantification relative to GAPDH (*n* = 3 replicates). *P* = 0.0475. **e** Representative staining for VP16 and gB in brainstems of HSV-1 infected *Optn*+/+ or −/− mice 8 dpi. Data are presented as mean values ± SEM (**b**, **d**). Two-tailed Student’s *t* test was performed for statistical analysis (*α* = 0.05). ^∗^*p* < 0.05; ns not significant. Source data are provided as a Source Data file.

**Fig. 3:**
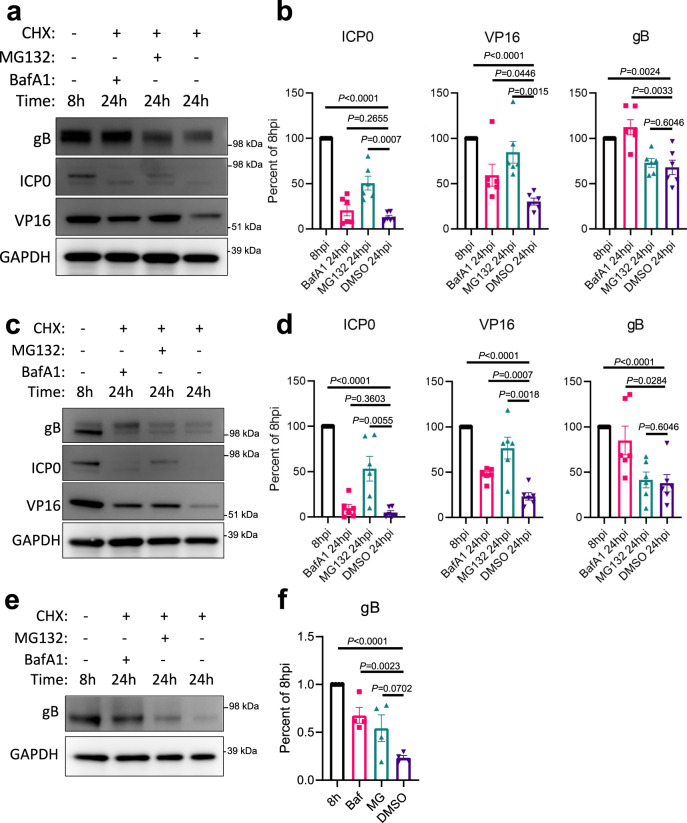
HSV-1 VP16 and gB, but not ICP0, are degraded in an autophagy dependent manner. Wildtype cells were infected at 1 MOI with HSV-1 17-strain for 8 h before CHX addition to block protein synthesis. In combination with CHX, either the autophagy inhibitor BafA1, the proteosome inhibitor MG132, or DMSO were added to cells. Cells were sampled at 8 h, and 24 h after infection. **a** Shown for HeLa cells are immunoblots against the HSV-1 proteins gB, ICP0, VP16, and the internal reference GAPDH, **b** with band quantification relative to GAPDH (*n* = 6 replicates). **c** Shown for HCE cells are immunoblots against the HSV-1 proteins gB, ICP0, VP16, and the internal reference GAPDH, **d** with band quantification relative to GAPDH (*n* = 6 replicates). Similarly, LUHMES cells were infected at 2.5 MOI with HSV-1 17-strain for 8 h before CHX addition to block protein synthesis. In combination with CHX, either BafA1, MG132 or DMSO were added to cells. Cells were sampled at 8 h, and 24 h after infection. **e** Shown for LUHMES cells are immunoblots against the HSV-1 protein gB and the internal reference GAPDH, **f** with band quantification relative to GAPDH (*n* = 4 replicates). Data are presented as mean values ± SEM (**b**, **d**, **f**). Two-tailed Student’s *t* test was performed for statistical analysis (*α* = 0.05). ^∗^*p* < 0.05; ^∗∗^*p* < 0.01; ^∗∗∗^*p* < 0.001, ^∗∗∗∗^*p* < 0.0001, ns not significant. Source data are provided as a Source Data file.

**Fig. 4:**
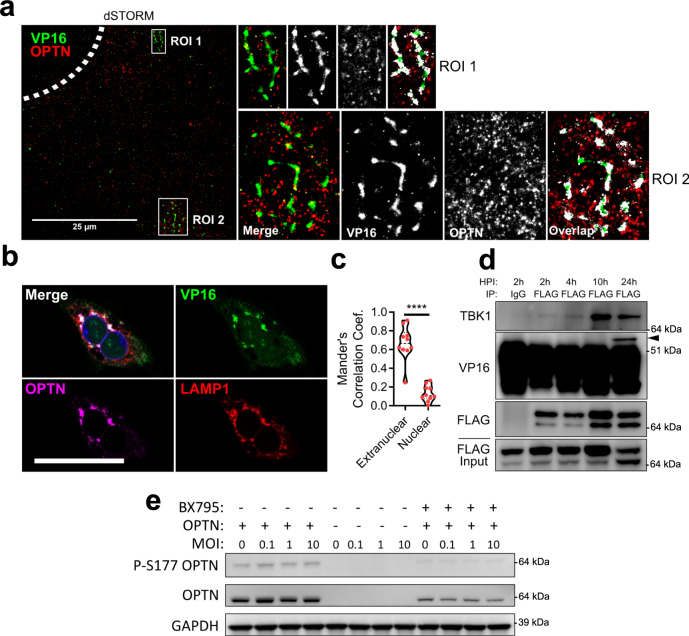
OPTN is associated with VP16 during HSV-1 infection and is activated by TBK1. **a** dSTORM super-resolution microscopy for OPTN and VP16 staining. Enlarged images highlight association of OPTN and VP16 stained structures. Shown to the right are the enlarged images for regions of interest (ROI) representing the merged images, the raw signal for VP16 and OPTN, and the pixel overlap between the channels. Scale bar is 25 µm. Dashed line represents nucleus. **b** Representative image of *Optn*+/+ cells infected with VP16-gfp HSV-1 at MOI 1 for 12 h stained for OPTN and LAMP1. Scale bar is 50 µm. **c** Mander’s colocalization coefficient representing the proportion of VP16 and OPTN co-staining. *n* = 10 cells analyzed for colocalization. *P* < 0.0001. **d** Immunoblot of anti-FLAG co-immunoprecipitation of *Optn*+/+ cells expressing FLAG-OPTN and infected with HSV-1 at 1 MOI. Arrow points to VP16 band. **e** Immunoblot of *Optn*+/+, *Optn*−/−, or *Optn*+/+ cells treated with TBK1 inhibitor, BX795 (50 µM), infected with HSV-1 for 3 h. Two-tailed Student’s *t* test was performed for statistical analysis (*α* = 0.05). ^∗∗∗∗^*p* < 0.0001. Source data are provided as a Source Data file.

OPTN has been established as an autophagy receptor for the selective autophagy of bacteria and organelles, therefore we hypothesized diminished viral protein levels due to selective autophagy against HSV-1. *Optn*+/+ or −/− cells were infected with HSV-1 for 12 h prior to addition of cycloheximide (CHX) to inhibit protein synthesis. *Optn*−/− cells expressed significantly higher levels of VP16 and gB, but not ICP0 (Fig. 2a, b). The levels of ICP0, VP16, and gB decreased over time for both genotypes despite the much higher levels of viral protein. This trend was the same for gB expression in HCE cells (Fig. 2c, d), and both gB and VP16 were detected in the central nervous system of HSV-1 infected *Optn*−/− mice but not *Optn*+/+ mice (Fig. 2e). To determine if the degradation of the proteins that were suppressed in an OPTN-dependent manner was autophagy dependent *Optn*+/+ cells were infected with HSV-1 for 8 h prior to the addition of CHX and either Bafilomycin A1 (BafA1) to inhibit autophagy, MG132 to inhibit the proteosome, or DMSO as a vehicle control. After 24 h of infection the 8 h starting level, 24 h with BafA1, and 24 h with MG132 protein levels were compared to the 24 hr DMSO levels. In *Optn*+/+ cells, ICP0 levels were significantly higher than DMSO treatment in the MG132 treatment group, VP16 levels were significantly higher than DMSO treatment in both BafA1 and MG132 treatment groups, and gB levels were significantly higher than DMSO treatment in the BafA1 treatment group (Fig. 3a, b). This trend was the same for HCE cells (Fig. 3c, d), and for the degradation of gB in LUHMES cells (Fig. 3e, f). This shows that degradation of gB and VP16 is autophagy dependent, and the levels of gB and VP16 are suppressed in an OPTN-dependent manner. Furthermore, super-resolution microscopy of cells infected with a GFP-VP16 fusion protein expressing HSV-1 strain revealed the association and staining overlap of OPTN stained aggregates with VP16 spherical structures which measured ~250 nm in diameter (Fig. 4a). Immunofluorescent staining also showed the strongly colocalized signal between OPTN, VP16, and the lysosome marker, lysosomal-associated membrane protein 1 (LAMP1), by which OPTN is associated with VP16 outside of the nucleus (Fig. 4b, c). TBK1 phosphorylation of OPTN at residue serine-177 is reported to regulate OPTN autophagy receptor activity^13^. Co-immunoprecipitation of OPTN showed that upon infection OPTN first interacted with TBK1, and then with VP16 (Fig. 4d). When TBK1 was inhibited using BX795, both the total level of OPTN and the phosphorylated form were decreased in an infection independent manner (Fig. 4e) suggesting that OPTN function is constitutively regulated by TBK1. This function of OPTN does not affect the total level of autophagy in HeLa or LUHMES cells as is reflected by the lack of increased or decreased LC3 lipidation, the process by which LC3 is recruited to autophagosomal membranes, during infection (Supplementary Fig. 3A–D). HSV-1 is known to inhibit host macroautophagy through the virulence factor, γ_1_34.5. While OPTN deficiency resulted in increased infection by wildtype HSV-1, infection with a γ_1_34.5 null mutant of HSV-1 resulted in equal levels of infection between both genotypes (Supplementary Fig. 3E). Together this data suggests that OPTN is a constitutively active antiviral defense factor that suppresses the expression of autophagy degradable essential viral proteins without changes to the total level of autophagy during infection.

### OPTN is neuroprotective against herpes simplex virus encephalitis

**Fig. 5:**
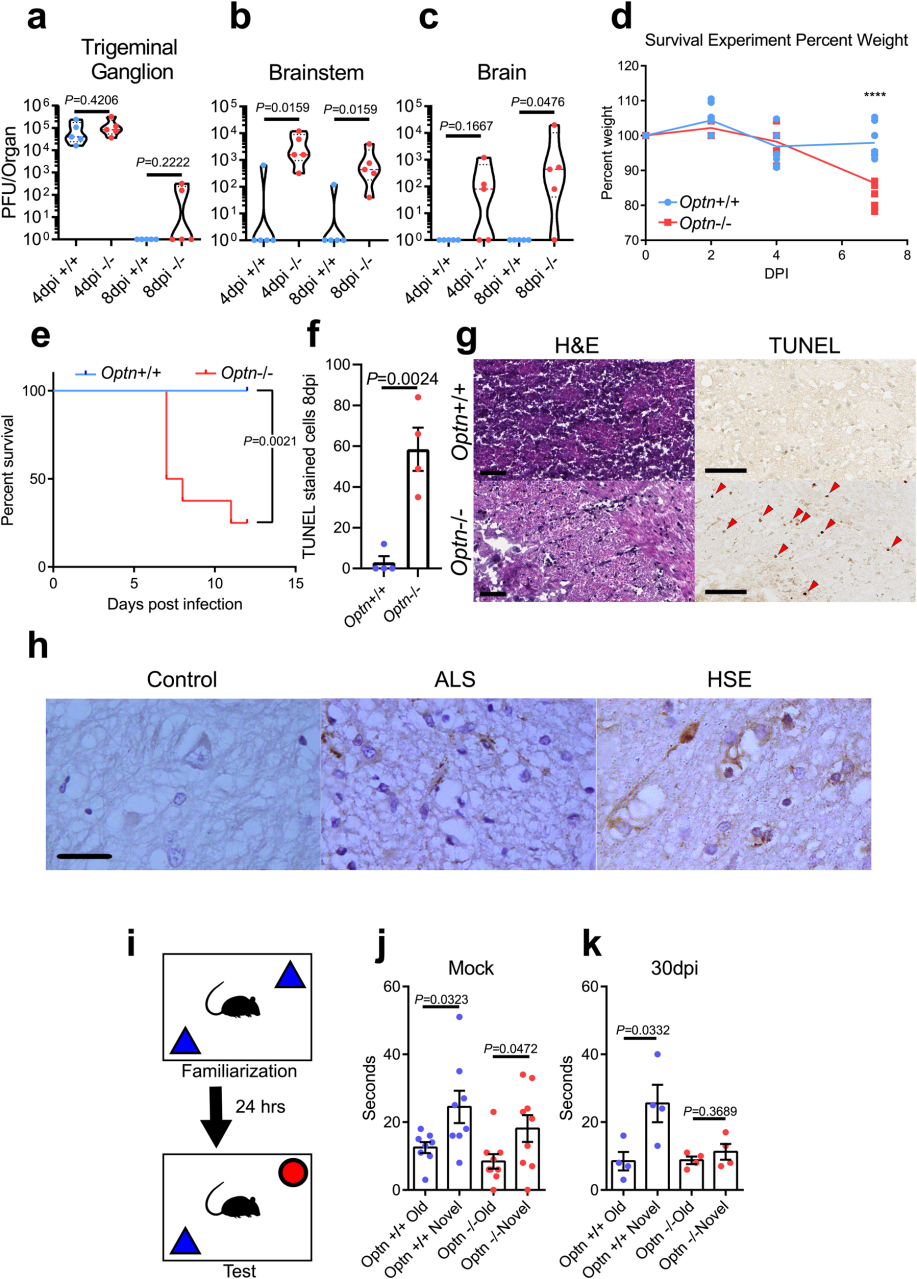
OPTN is neuroprotective against herpes simplex virus encephalitis. Mice were infected using corneal scarification using 5 × 10^5^ PFU of HSV-1 McKrae strain. **a**–**c** Tissue plaque assay for trigeminal ganglion (left), brainstem (middle), and brain (right) (*n* = 5 mice per group). Two-tailed Mann–Whitney *U* test was used where *p* < 0.05 indicates significance. **d** Weight changes of mice (*n* = 5 mice per group) up to 7dpi. Two-way repeated-measures ANOVA *F*_(3,42)_ = 9.908, *p* < 0.0001. **e** Kaplan–Meier curve for HSV-1 infected *Optn*+/+ (*n* = 8) and −/− (*n* = 8) mice. Logrank test was used to compare curves with *p* < 0.05 being significant. **f**, **g** (left) Quantitation of TUNEL stained cells in brainstems of *Optn*+/+ and −/− mice 8dpi (*n* = 4 mice per group). (right) Representative images of H&E staining and TUNEL staining (dark brown) for brainstems. Arrows highlight TUNEL positive cells. Scale bar is 50 µm. **h** Immunohistochemistry staining of OPTN (dark brown) in human brainstem tissue from a healthy, amyotrophic lateral sclerosis (ALS), or HSE patient shows aggregation of OPTN protein expression in diseased tissues. Scale bar is 100 µm. **i** Schematic of Novel Object Recognition (NOR) test for mice. **j** mock-infected *Optn*+/+ and −/− mouse exploration times for old and novel objects. (*n* = 8 mice per group) (**k**) *Optn*+/+ and −/− mouse exploration times for old and novel objects at 30 dpi with 1 × 10^5^ PFU of HSV-1 McKrae strain (*n* = 4 mice per group). Data are presented as mean values ± SEM (**f**, **j**, **k**). Unless otherwise indicated, Two-tailed Student’s *t* test was performed for statistical analysis (*α* = 0.05). ^∗^*p* < 0.05; ^∗∗^*p* < 0.01; ^∗∗∗∗^*p* < 0.0001, ns not significant. Source data are provided as a Source Data file.

To confirm the relevance of our in vitro results, we used an *Optn*−/− mouse model for HSV-1 infection. Infection of *Optn*+/+ and *Optn*−/− mice revealed striking differences in severity of infection. The infection spread to the trigeminal ganglion (TGN) of both mouse strains, but significant infection of the brainstem and brain only occurred in *Optn*−/− mice as observed by tissue viral titers (Fig. 5a–c). *Optn*−/− mice experienced significant decrease in weight and increased mortality compared to *Optn*+/+ mice (Fig. 5d, e). Neuron death was nearly undetectable in *Optn*+/+ brainstem tissue, but was high in *Optn*−/− tissue, indicating that OPTN protects the CNS from HSV-1 neuro-invasion and neurodegeneration during acute infection (Fig. 5f, g). Human brainstem tissue from a normal brain, an ALS patient, and a herpes simplex virus encephalitis (HSE) patient were stained for OPTN (Fig. 5h). OPTN aggregates were absent in the control tissue but were present in the ALS tissue which is consistent with previously published reports^17,18^. In the HSE tissue we found widespread OPTN stained cytosolic aggregates, implicating a similar pathology in herpetic human diseases. Ultimately the damage to neurons in *Optn*−/− mice resulted in behavioral indication of neurodegeneration. In a novel object recognition test, *Optn*−/− animals showed a preference to explore a novel object without infection, but 30dpi *Optn*−/− animals did not exhibit the same preference, which is a known indicator for loss of cognition related to memory (Fig. 5i–k)^27–29^.

### OPTN deficiency impairs host immunity

**Fig. 6:**
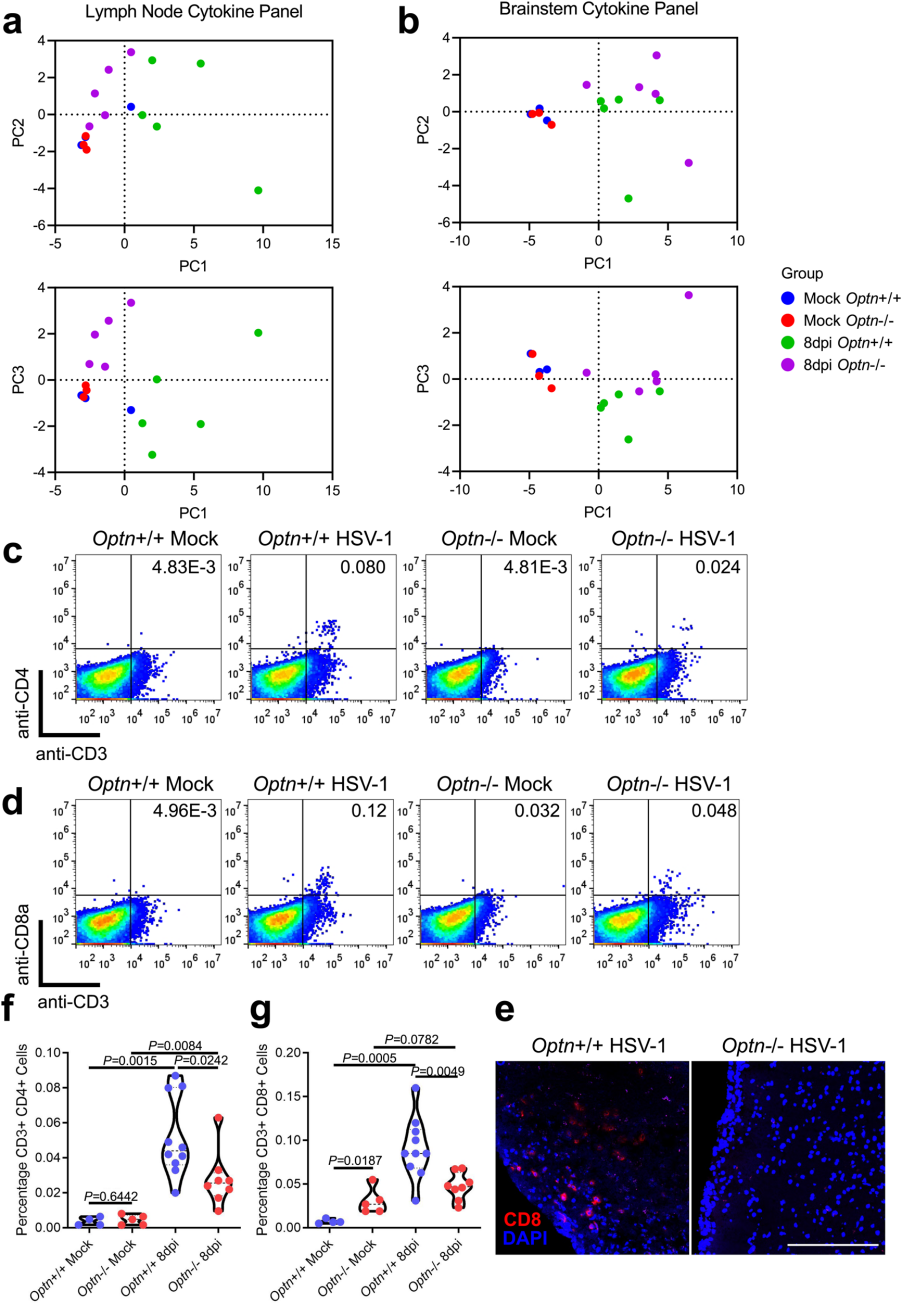
OPTN deficiency impairs recruitment of CD4 and CD8 lymphocytes to the CNS. Principal component analysis of cytokine protein expression profiles from 8dpi (*n* = 5 mice per group) or mock infected (*n* = 3 mice per group) *Optn*+/+ or −/− mice to compare (top) PC1 vs PC2 or (bottom) PC1 vs PC3 are shown for **a** draining lymph nodes and **b** brainstems. Brainstem cell suspensions from mock infected *Optn*+/+ (*n* = 4 mice), mock infected *Optn*−/− (*n* = 5 mice), 8dpi *Optn*+/+ (*n* = 10 mice), and 8 dpi *Optn* −/− (*n* = 8 mice) were analyzed using flow cytometry to measure the percentage of T cells present. **c** Dot plots representing CD3 + CD4 + cells. **d** Dot plots representing CD3 + CD8 + cells. **f** Quantification of percentage CD3 + CD4 + cells. **g** Quantification of CD3 + CD8 + cells. **e** Staining for CD8 in 8 dpi mouse brainstem tissue. Scale bar is 200 µm. Two-tailed Student’s *t* test was performed for statistical analysis (*α* = 0.05). ^∗^*p* < 0.05; ^∗∗^*p* < 0.01; ^∗∗∗^*p* < 0.001, ns, not significant. Source data are provided as a Source Data file.

Immunity to HSV-1 depends partly on recruitment of CD8 T cells to the site of infection^7^. Through cytokine profiling we revealed dramatic differences in the inflammatory state of *Optn−/−* mice. The *Optn*−/− draining lymph nodes (dLNs) had less pro-inflammatory cytokine expression apart from IFNγ, and the overall cytokine profiles diverged between the two genotypes (Fig. 6a and Supplementary Fig. 4A). This is reflected in the lack of lymphadenopathy in the *Optn*−/− mice (Supplementary Fig. 4C, D). There was more expression of proinflammatory markers in *Optn*−/− mouse brainstems, suggesting greater neuroinflammation (Fig. 6b and Supplementary Fig. 4B). This was accompanied by fewer CD3+CD8+ and CD3+CD4+ cells in brainstems of *Optn*−/− animals during infection (Fig. 6c–e). Despite the increased viral load in the *Optn*−/− mice, they had a stunted immune response through poor dLN activation and diminished T cell recruitment to the site of infection. Curiously, there was increased expression of IFNγ in the *Optn*−/− mice in both tissues examined.

### OPTN suppresses necroptosis in vitro and in neurons during HSV-1 infection

**Fig. 7:**
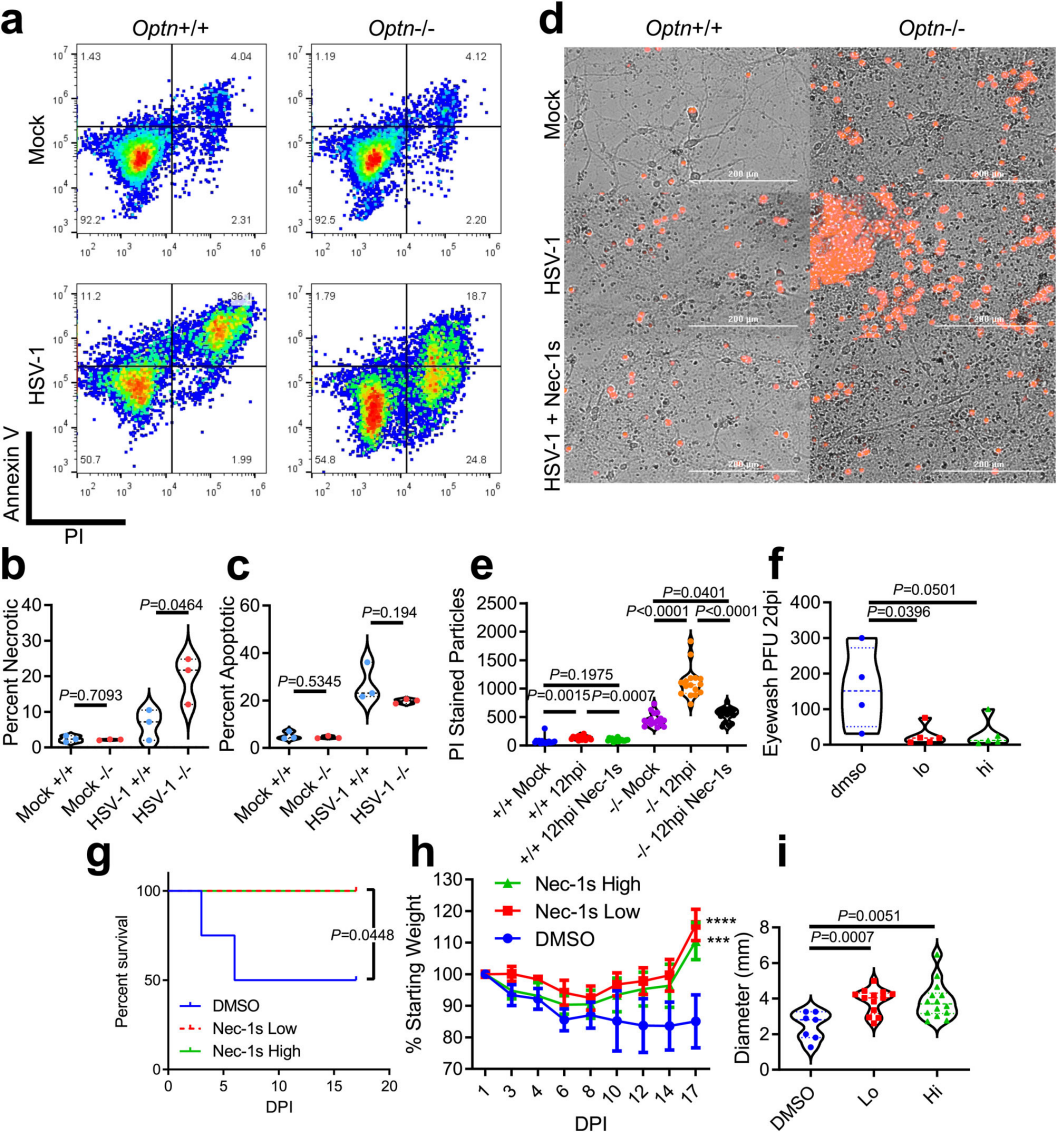
OPTN suppresses necroptosis in vitro and in neurons during HSV-1 infection. **a** Dot plots of flow cytometry analysis of *Optn*+/+ and −/− cells stained for Annexin V and propidium iodide (PI). **b**, **c** Percentage of (**b**) Annexin V lo, PI hi cells indicating necrotic cells or **c** Annexin V hi, PI hi cells indicating apoptotic cells. **d** Representative images of primary embryonic neurons from *Optn*+/+ or −/− mice that were mock infected, HSV-1 infected, or HSV-1 infected and Nec-1s treated. Dead cells were stained using PI (Red). **e** Image quantification of PI-stained primary neurons. **f**–**i** *Optn*−/− were treated with hi Nec-1s (*n* = 5 mice), lo Nec-1s (*n* = 5 mice), or DMSO (*n* = 4 mice) during infection. **f** Plaque assay on ocular washes from *Optn*−/− mice 2 dpi. **g** Kaplan–Meier curve for HSV-1 infected *Optn*−/− treated with DMSO, lo or hi Nec-1s. Logrank test for trend was used to compare curves with *P* < 0.05 being significant. **h** Percentage of initial weight over time. Two-way repeated-measures ANOVA *F*_(16,88)_ = 1.758, *p* = 0.0503, multiple comparisons at 17 dpi (DMSO vs. Nec-1s Low) *p* < 0.0001, (DMSO vs. Nec-1s High) *p* = 0.0009. **i** dLN diameters for *Optn*−/− mice treated with DMSO, lo Nec-1s, or hi Nec-1s at 17dpi and data are presented as mean values ± SEM. Unless otherwise stated Two-tailed Student’s *t* test was performed for statistical analysis (*α* = 0.05). ^∗^*p* < 0.05; ^∗∗^*p* < 0.01; ^∗∗∗^*p* < 0.001, ^∗∗∗∗^*p* < 0.0001, ns not significant. Source data are provided as a Source Data file.

Necroptosis is suggested to be an antiviral response, but also a cause of neurodegeneration when it occurs in neuronal tissues^24^. OPTN negatively regulates necroptosis by targeting RIPK1 for autophagic degradation^24^. Pharmacological inhibition of autophagy or single gene knockout of essential autophagy genes, *Atg5*, *Fip200*, *Atg16l1*, or *Becn1* are reported to protect cells from necroptosis mediated through IFNγ, TNF-α, and RIPK1^25^. Having shown that CNS infection leads to neuron death, and IFNγ was highly represented in multiple tissues of *Optn*−/− mice, we sought to understand the role of OPTN in necroptosis during infection. Annexin V and propidium iodide staining revealed an OPTN-dependent divergence in cell death pathways, whereby OPTN removal shifted an apoptotic response to a necrotic response (Fig. 7a–c). RIPK1 inhibitor, Necrostatin-1s (Nec-1s), rescued *Optn*−/− primary neurons from HSV-1-induced death (Fig. 7d, e). Similar results were observed in vivo where Nec-1s treatment rescued *Optn*−/− mice survival, weight, viral load, and lymphadenopathy (Fig. 7f–i). These results confirmed that the increased cell death was RIPK1-dependent which is a hallmark of necroptosis^24^. Our findings suggest excess necroptosis may not be a beneficial innate defense mechanism during HSE and may be deleterious instead. It is interesting that inhibition of RIPK1 recovered the lymphadenopathy of *Optn*−/− animals, and may indicate there is OPTN-dependent crosstalk between the IFNγ, TNF-α, RIPK1 axis, and autophagy machinery during infection^25^.

## Discussion

Previous reports outline mechanisms by which organelles and bacteria are degraded through OPTN-mediated selective autophagy^13–15^. We have shown that these mechanisms also apply to HSV-1 infection, revealing what is likely an evolutionarily conserved anti-herpesvirus intrinsic cellular defense. OPTN is regulated by TBK1 during infection, as inhibition of TBK1 inhibits the expression and phosphorylation of OPTN during HSV-1 infection. We also demonstrated the increased association of OPTN and TBK1 during infection, which was followed by association of OPTN and VP16. Additionally, we show that OPTN suppression of HSV-1 protein expression is selective for autophagy degradable viral factors, as gB and VP16 expression were significantly lower in wildtype cells, but ICP0 expression was unaltered. Additionally, we observed that MG132 also resulted in significant inhibition of VP16 degradation, suggesting there may be multiple pathways involved in the degradation of VP16 during infection. gB is an essential glycoprotein present on the envelope of HSV-1 virions, and is responsible for fusion with host cell membranes upon entry^30^. VP16 is a transactivating factor that hijacks host transcription factors to promote transcription of viral genes during the lytic cycle of infection^31^. This restricted the spread of HSV-1 and PRV in different cell lines.

In a report published during this study, OPTN was shown to be downregulated as a potential strategy to enhance HSV-1 infection, but OPTN knockdown did not result in increased HSV-1 infection in human embryonic lung fibroblasts^32^. We have shown in HCE cells and LUHMES differentiated into neurons that OPTN knockdown leads to enhanced infection. Additionally, our in vitro and in vivo knockout models showed increased infection by HSV-1. This is the first demonstration that OPTN-mediated autophagy is involved in degradation of HSV-1 particles and has an antiviral role. Interestingly, this is in contrast with the pro-viral role reported elsewhere for RNA viruses^23^. However, unlike our study, the RNA virus study did not use knockout in vitro and in vivo models to arrive at their conclusions. OPTN may have different effects in response to viruses ranging from restrictive to permissive depending on the lifecycle and host-interactions of the virus in question. Additionally, other autophagy receptors may demonstrate similar anti-viral capabilities during HSV-1 infection.

Despite the role of OPTN in reducing HSV-1 protein expression and restricting spread of infection, OPTN does not affect the total level of autophagy within cells during infection of the knockdown or knockout in vitro models. Unexpectedly, the presence of OPTN had no effect on the level of replication of a γ_1_34.5 null HSV-1 strain. Regardless of starting protein level, cells were able to degrade VP16 and gB proteins upon cycloheximide addition and the total level of LC3 lipidation was unchanged. We interpret these observations to suggest that OPTN selective targeting of viral proteins is not crucial when γ_1_34.5 is unable to inhibit host autophagosome formation. This might be because there are potentially more available autophagosomes to compensate for lack of OPTN. Further study is required to better understand the interaction of γ_1_34.5 and OPTN.

Beyond degrading HSV-1 protein, we show that OPTN is closely linked with optimal immune function and neuronal survival. OPTN has been shown to regulate interferon induction, and TNFR (TNF-α receptor) signaling to the benefit or detriment of cells^21–23^. Much previous work on OPTN-dependent regulation of cytokine signaling has been performed in vitro, but our in vivo data shows tissue specific differences in cytokine profiles between the *Optn*+/+ and −/− mouse strains. In the dLNs, there was enhanced immune activation when OPTN was present, but the CNS tissue revealed the opposite trend where IFN-γ was highly represented in OPTN-deficient mice. This shows there are further tissue specific determinants of cytokine signaling which interact with OPTN to drive or negate cytokine production such as cell types, local immunity, or permissiveness to HSV-1 infection, and these determinants are linked to selective autophagy^21–23^.

Downstream of TNFR stimulation, OPTN selectively degrades RIPK1, a necroptosis signaling molecule implicated in increased chronic CNS necroptosis^24^. Previous reports have implicated OPTN deficiency as a mechanism for the progression of ALS^24^. We have shown that acute CNS necroptosis can occur during HSV-1 infection in *Optn−/−* mice, leading to encephalitis, long-term neurodegeneration, and death, further supporting the anti-viral restriction by OPTN. This is rescuable by treatment with the RIPK1 inhibitor, Nec1s. Nec1s may be a potential drug candidate to treat viral encephalitis, but the efficacy needs to be directly assessed before use outside of animal models.

Lastly this study focused on the role of OPTN in protection of the CNS, but the study has not addressed the role of OPTN in the peripheral nervous system (PNS). In both the *Optn*+/+ and −/− mice the infection reached the trigeminal ganglion but failed to spread to the CNS of *Optn*+/+ mice. HSV-1 treatments and pathologies are complicated by the ability of herpesviruses to establish latency. OPTN suppresses the expression of VP16, a key protein in establishment and reactivation from latency. OPTN suppression of VP16 might prevent HSV-1 reactivation from latency because VP16 is a transactivating factor that can drive expression of a lytic gene program in sensory neurons^33,34^. Future work is required to investigate the relationship between OPTN and latency.

Selective autophagy has emerged as an important cellular process for maintaining the health of neuronal tissues and resisting neurodegeneration. Dysfunction in OPTN is implicated in glaucoma and neurodegenerative diseases including ALS and AD^5,18^. Viral infection of the CNS has been hypothesized to contribute to the etiology of neurodegenerative disorders, and defects in autophagy may synergize with infection and subclinical neuroinflammation to accelerate neurodegeneration^1–3^. We show that mice lacking OPTN demonstrate accelerated decline in cognitive functions following HSV-1 infection, underscoring the role of OPTN and HSV-1 in the potential etiology of neurodegeneration with cognitive decline. Many viruses encode virulence factors that alter host autophagy machinery, and despite the rarity of viral encephalitis, many individuals are seropositive for viruses with this capability^4^. Taken together we have shown the necessity of OPTN-mediated selective autophagy in targeting of HSV-1, recruitment of wider immune responses, and protecting the CNS from neurotropic viral infection and neuronal cell death. Our study demonstrates that defects in OPTN functions compounded by HSV-1 infection have the potential to cause accelerated neuronal damage.

## Methods

### Antibodies, stains, cells, viruses, chemicals, and plasmids

The following antibodies and stains were used in this study for imaging:

DAPI (D9542, Sigma) (1:1000), NucBlue™ Live ReadyProbes™ Reagent (Thermo Fisher, R37605) (2 drops per ml), Mouse monoclonal to LAMP1 (Abcam, ab25630, [H4A3]) (1:100), Mouse monoclonal anti-HSV1 + HSV2 VP16 (Abcam, ab110226, [LP1]) (1:100), Goat anti-Mouse IgG (H + L) Highly Cross-Adsorbed Secondary Antibody, Alexa Fluor 546 (Thermo Fisher, A-11030) (1:100), Goat anti-Rabbit IgG (H + L) Highly Cross-Adsorbed Secondary Antibody, Alexa Fluor 647 (Thermo Fisher, A-21245) (1:100), Rabbit polyclonal anti- Optineurin (CTerm) (Cayman Chemical, No. 100000) (1:100), and anti-mouse CD8a APC-conjugated monoclonal antibody (Tonbo biosciences, 20-1886-U100, [clone 2.43]) (1:100).

The following antibodies were used for immunoblot:

Mouse monoclonal anti-GAPDH (Santa Cruz, sc-69778, [7B]) (1:1000), Goat anti-Mouse IgG (H + L) Highly Cross-Adsorbed Secondary Antibody HRP (Thermo Fisher, 31432) (1:5000), Goat anti-Rabbit IgG (H + L) Cross Adsorbed Secondary Antibody HRP (Thermo Fisher, G-21234) (1:5000), Mouse monoclonal anti-FLAG (Sigma, F1804, [M2]) (1:1000), Mouse monoclonal anti-HSV1 ICP0 (Abcam, ab6513, [5H7]) (1:1000), Rabbit monoclonal anti-p-S177 OPTN (Cell Signaling Technologies, 57548 S) (1:1000), Mouse monoclonal anti-HSV1 + HSV2 VP16 (Abcam, ab110226, [LP1]) (1:1000), and Rabbit polyclonal anti- Optineurin (CTerm) (Cayman Chemical, No. 100000) (1:1000).

The following antibodies were used for immunoprecipitation:

Mouse monoclonal anti-FLAG (Sigma, F1804, [M2]) (5 µg), normal mouse IgG (Santa Cruz, sc-2025) (5 µg), and additionally, and Protein A/G PLUSAgarose beads (Santa Cruz, sc-2003) (20 µl) were used.

For flow cytometry the following antibodies were used:

anti-mouse CD8a APC-conjugated monoclonal antibody (Tonbo biosciences, 20-1886-U100, [clone 2.43]) (1:100). anti-mouse CD4 PE-conjugated monoclonal antibody (Tonbo biosciences, 50-0042-U100, [RM 4-5]) (1:100).

HeLa *Optn*−/− and parental strain were provided by Dr. Richard Youle (National Institutes of Health). Vero cells (ATCC) were used in plaque assays and in virus preparation. LUHMES cells (ATCC) were provided by Dr. David Bloom (University of Florida). HCE cells were provided by Dr. Kozaburo Hayashi (National Eye Institute, Bethesda, MD). Paul Kinchington (University of Pittsburgh) provided the dual tagged KOS strain of HSV-1 (RFP driven by gC promoter and GFP driven by ICP0 promoter).

Dr. Patricia Spear (Northwestern University) provided the KOS and 17 strains. The strain 17 γ_1_34.5 -null mutant of HSV-1 was provided by Dr. David Leib (Dartmouth College). Dr. Prashant Desai (Johns Hopkins University) provided the HSV-1 K26-GFP KOS strain. Dr. Steven Triezenberg (Van Andel Research Institute) provided the DG1 (VP16-GFP expressing) strain of HSV-1. Gregory Smith (Northwestern University) provided the PRV-GS2484 strain (PRV-Becker expressing mRFP1-UL35 fusion protein and CMV-GFP cassette in Us4).

The following chemicals were used in this study: Bafilomycin A1 (Sigma) to inhibit autophagic flux, MG132 (Sigma) to inhibit proteasomal degradation. Cycloheximide (Sigma) to block de novo protein synthesis. Lipofectamine 2000 (Thermo Fisher) and RNAiMAX (Thermo Fisher) for transfections. Necrostatin-1s (Selleckchem) for inhibition of necroptosis. BX795 (Selleckchem) for inhibition of TBK1.

The FLAG-OPTN plasmid was provided by Dr. Beatrice Yue (University of Illinois at Chicago).

### Mice

Mice used in this study were generated in the study^35^. In summary *Optn*^flox/flox^ mice on a C57BL/6 background were generated using a targeting vector-inserted LoxP site that flanks the first coding exon and a neomycin selection cassette. *Optn*^flox/flox^ mice were crossed with CMV-Cre mice (Jackson Laboratories, Bar Harbor, ME, USA) to generate CMV-Cre; *Optn*^flox/wt^ mice, which were used as breeding pairs. Global *Optn* knockout (CMV-Cre; *Optn*^flox/flox^) mice are hereafter referred to as *Optn* − /− mice. 8-16 weeks old *Optn* − /− or *Optn*+/+ male and female mice were used in this study.

All experiments were performed and housed in a BSL2 rodent facility in the Biologic Resources Laboratory at the University of Illinois at Chicago with a standard 12 h light, 12 h dark cycle. Ambient temperatures at this facility are maintained between 20 and 26 °C and relative humidity between 45% and 65%. This modern animal facility has several veterinarians on staff available for expert veterinary care and advice during the project. Animal services core facility at the Department of Ophthalmology and visual sciences houses a BSL-2 facility dedicated specifically to our laboratory use. All protocols have been reviewed and certified by the animal care committee of the University of Illinois at Chicago.

### Mouse infection experiments

The corneal scarification method was used for infection of mice. Mice were anesthetized using a mixture of ketamine and xylazine, and the topical anesthetic, proparacaine, was applied to the ocular surface prior to epithelial debridement. Three parallel scratches were made in the corneal epithelium with a 30-gauge needle, then mice were infected with 5 × 10^5^ PFU HSV-1 by application of a virus containing solution to the corneal surface. For the novel object recognition test mice were infected with 1 × 10^5^ PFU HSV-1 to reduced number of mice that reached endpoint criteria before 30 dpi. McKrae strain was used for mouse experiments. For survivorship endpoint criteria, mice had to lose >15% initial body weight rapidly, demonstrate excessive morbidity, difficulty ambulating, paralysis, or deep lesions on the head > 1cm^2^.

### Novel Object Recognition Test

The Novel Object Recognition Test was be used to assess whether deterioration of memory is occurring in mice during ocular HSV-1 infection. Mice were 8-16 weeks old. The apparatus for testing was a white polypropylene box with a video recording camera (GoPro Hero7, GoPro) mounted above it. The base of the box is 15 inches by 20 inches and the walls of the box are 11.75″ inches high. The test for each treatment group included a familiarization session and a testing session, both of which were recorded.

The familiarization session began with cleaning the box and objects with MB10 to both disinfect the area and minimize odor cues. Each animal was subjected to a familiarization session where two identical objects were placed in the box prior to introducing the mouse to the box. The mouse was introduced to the box and allowed to explore the box for a total of 5 min. At this point the mouse was removed from the testing area and the area and objects were cleaned again with MB10 before testing the next mouse.

24 h later each animal was subjected to a testing session where two objects were placed in the box prior to introducing the mouse to the box. One object was from the familiarization session, and the second was a novel object. The session began with cleaning the box and objects with MB10 to both disinfect the area and minimize odor cues. The mouse was introduced to the box and allowed to explore the box for a total of 5 min. At this point the mouse was removed from the testing area and the area was cleaned again with MB10 before testing the next mouse.

Using the recorded footage, the time the mouse explored each object was measured manually using a stopwatch. Only exploration including closely sniffing or touching the object with whiskers or nose was counted as exploration.

### Necrostatin-1s experiments

For mouse experiments, Nec-1s was administered at a low dose of 0.5 mg/kg (lo), high dose of 2.5 mg/kg (hi), or control treatment of vehicle alone (DMSO). Administration by intraperitoneal injection was performed 12 h before infection (0dpi), then daily until 7 dpi. For in vitro experiments 15 days in vitro (DIV) primary neuron culture from *Optn*+/+ or *Optn*−/− mouse embryos were infected with MOI 2.5 17 strain HSV-1 for 1 h before medium was changed. The replacement medium included 1 µg/ml propidium iodide (Invitrogen) and either 100 µg/mL Nec-1s or DMSO. Medium was used for the remainder of the experiment and images were captured using a Lionheart LX automated microscope.

### BX795 experiments

10^6^ HeLa *Optn*+/+ or −/− cells were plated in six-well format. The next day one group of wells were treated with 50 µM BX795 for 1 h then all groups were infected at indicated MOI with 17 strain HSV-1 for 3 h. Samples were harvested for immunoblotting.

### Cycloheximide chase experiments

10^6^ HeLa *Optn*+/+ or −/− cells were plated in six-well format, or 5 × 10^5^ HCE cells were plated in 12-well format and transfected with siRNA. The next day cells were infected at MOI 1 with HSV-1 (17-strain) for 12 h. Medium (DMEM 10% FBS for HeLa, MEM 10% FBS for HCE) was replaced with fresh medium containing 100 µg/mL cycloheximide to block de novo protein translation. Samples were collected at 12 h, 18 h, and 24 h after infection for lysis in RIPA buffer and subsequent immunoblotting. In a separate experiment using only wildtype HeLa, LUHMES, or HCE cells, at 8 hpi fresh medium containing 100 µg/ml cycloheximide to block de novo protein translation and either DMSO, as a control, 200 nM Bafilomycin A1 to inhibit autophagy, or 50 µM MG132 to inhibit the proteasome. An untreated sample was taken at 8 hpi, and treated samples were taken at 24 hpi for immunoblotting.

### siRNA transfection

A Dicer-Substrate Short Interfering RNAs (DsiRNAs) TriFECTa® Kit (IDT) with predesigned siRNA molecules was used for transfections in this study. Cells were plated and grown to 50% confluency. Cells were then transfected as per manufacturer’s protocol using RNAiMAX at 1 µl/mL in OptiMEM (ThermoFisher). Multiple concentrations for each premade siRNA molecule were tested and it was determined that siRNA 1 at 1 nM produced effective knockdown with minimal cell death after 48 h of transfection.

### LUHMES cell culture

LUHMES cell culturing was adapted from the methods outlined^36^. First tissue culture flasks and plates were coated with poly-l-ornithine hydrobromide (Sigma) overnight in a biosafety cabinet then with fibronectin (Sigma) overnight in a tissue culture incubator at 37˚C. Plates were allowed to air dry before use. Proliferation medium (DMEM:F12 (ATCC) containing 1% N2 Supplement (ThermoFisher), 1X Penicillin-Streptomycin-Glutamine solution (ThermoFisher), and recombinant human FGF-basic (Fibroblast Growth Factor, Peprotech) was prepared immediately before use and was used to passage the undifferentiated LUHMES cells. Cells were plated in plates and grown to 50% confluency before differentiating in differentiation medium with 1 µg/mL tetracycline hydrochloride (Sigma), 1 mM N6,2′-O-Dibutyryladenosine 3′,5′-cyclic monophosphate sodium salt (Sigma), and 2 ng/mL Glial cell-derived neurotrophic factor (GDNF). In experiments where siRNA transfection was required, the transfection was performed overnight in OptiMEM (see siRNA transfection section) prior to addition of differentiation medium. Cells were then used for experiments 4 days after differentiation.

### Primary neuron culture

Embryonic brains from E18 mouse embryos were pooled for each mouse strain and digested in 5 mL 0.25% Trypsin with 75 µL 0.1% DNase (New England Biolabs) in a 37 °C water bath for 30 min. Tissue was spun down at 500 × *g* for 1 min to aspirate trypsin and wash pellet with 5 mL cFBS (Gibco). Cells were spun down and resuspended in 5 mL medium (485 ml Neurobasal®-A medium (Gibco), 10 mL B27 supplement (Invitrogen), 5 mL 200 mM L-glutamine (Invitrogen), 25 mM glucose). Tissue was triturated with a 5 mL plastic pipette for 10 repetitions, then with a 1000 µL pipette for 10 repetitions before being filtered through a 70 μ strainer into a new 50 mL conical tube and spun down. The medium was aspirated and the cells were resuspended in 20 mL medium. In all, 0.5 × 10^6^ cells were plated in 1 mL medium into Poly-d-Lysine coated 24-well plates. At 3 DIV a half medium change with Cytosine β-d-arabinofuranoside hydrochloride (Ara-C) containing medium to a final concentration of 1 µM Ara-C. Subsequent restorative half medium without Ara-C were performed at DIV6, DIV8, DIV10, and DIV13. Experiments were performed on DIV15.

### Viral genome isolation

Infected cell pellets were suspended in buffer containing 1% SDS, 50 mM Tris (pH 7.5), and 10 mM EDTA, and proteinase K (2 units/mL) then incubated overnight at 45 °C. Proteinase K is then heat inactivated for 30 min at 95 °C. DNA was isolated using phenol/chloroform extraction followed by ethanol precipitation. HSV-1 genomes were quantified using quantitative PCR (ABI 7500, Applied Biosystems) using HSV-1 specific primers and Fast SYBR Green Master Mix (Life Technologies). Primers are listed in Supplementary Table 1.

### Time-lapse fluorescent microscopy

Immediately after the addition of HSV-1 in cell growth medium (DMEM, 10% FBS, 1% Penn/Strep), cells were placed in the environmentally controlled chamber on the stage of a Zeiss Observer spinning disk confocal microscope set to 37 °C and 5% CO_2_. Time-lapse experiments images were taken at 30 min intervals or 60 min intervals. Imaging was performed using either 10x or 100x objective lenses. Image analyses were performed in ZEN and imageJ software.

### Fluorescent microscopy

Cells grown on 1.5 mm thick glass bottom dishes were fixed in 4% paraformaldehyde for 10 min. If antibody staining was performed, fixation was followed by permeabilization in a 0.1% Triton X 100 solution. Samples were blocked in 5% fetal bovine serum in phosphate-buffered saline (blocking buffer) for 1 h. Samples were stained with primary antibody (1:100) in blocking buffer for 1 h followed by secondary antibody (1:100) and DAPI staining for 1 h. Images were taken on a Zeiss LSM710 confocal microscope using 63x or 100x objective lenses. For the mouse brain sections, additional images were captured on a BioTek Lionheart microscope at ×10 and image-stitched using the Biotek GEN5 v3.04 Imager software.

### TIRF/super-resolution microscopy

Following the same staining protocol for fluorescent microscopy with two modifications; cells were washed five times with blocking buffer after each staining, and antibody concentrations were doubled (1:50) with DAPI omitted. Imaging was performed using a DMi8 S platform equipped for TIRF and ground state depletion (GSD) (Leica). OxEA buffer, an oxygen scavenger buffer, must be made immediately before use and is exhausted after ~1 h of imaging. OxEA (50 mM β-MercaptoEthylamine hydrochloride [Sigma], 3% v/v OxyFluor [Oxyrase Inc], 20% v/v sodium dl-lactate solution [Sigma], in PBS pH 8-8.5 adjust with NaOH) was added to the fixed, permeabilized, and stained cells prior to imaging. Once a location of interest was TIRF-imaged using the LAS X software to calculate and adjust the machine for a 250 nm depth image, GSD was used starting with the longest excitation wavelength and ending with the shortest to collect 5000 images of “blinking” fluorescence for each channel. Post-imaging dSTORM reconstruction in FIJI with the ThunderSTORM plugin was performed to acquire super-resolution images.

### Immunohistochemistry

Tissue was embedded in OCT Compound (Thermo Fisher Scientific) and frozen. 10 µm sections were cut from the tissue in a CryoStar NX50 (Thermo Fisher Scientific) cryotome and collected on double frosted slides (Thermo Fisher Scientific). Slides were fixed in cold 100% acetone for 10 min. Slides are then washed three times in phosphate-buffered saline with tween-20 (PBST). Slides were blocked in 5% Bovine Serum Albumin in PBS for 30 min at room temperature. Slides were then stained with the primary antibody (1:100 in PBST) for 60 min at room temperature followed by three washes in PBST. Protected from light, slides were stained with the secondary antibody (1:100 in PBST) and DAPI for 60 min at room temperature. Slides were washed three more times in PBST then mounted Vectashield hardset mounting medium (Vector Laboratories, Burlingame, CA, United States), sealed with clear nail polish, then visualized by fluorescence microscopy. For TUNEL staining, cyrosections of mouse brain tissue was stained using a TUNEL Assay Kit - HRP-DAB (ab206386) (Abcam) according to manufacturer’s protocol. For HSV-1 VP16 and gB stainings, the M.O.M.® (Mouse on Mouse) Immunodetection Kit, Fluorescein (FMK-2201) (Vector Laboratories, Burlingame, CA, United States) was used according to manufacturer’s protocol.

### Immunohistochemical detection of optineurin in human nervous systemtissues

Human nervous system tissues analyzed by immunohistochemistry for optineurin expression were derived from autopsies performed at the University of Illinois at Chicago. Autopsies were obtained after informed consent and tissue use conformed to Institutional Review Board (IRB)-approved protocols. Sections of paraffin-embedded human brain tissues were prepared and evaluated using standard histopathologic techniques and neuropathologic diagnostic criteria by a board-certified neuropathologist. For immunohistochemical analysis, 5 µm sections of paraffin-embedded formalin fixed tissue were deparaffinized with xylene and rehydrated. Antigen unmasking was performed in 1X citrate buffer, pH 6.0 just below boiling for 10 min. Reaction container and slides were allowed to cool for 20 min. The slides were then removed from reaction container, washed in dH2O, incubated in 3% hydrogen peroxide for 10 min, washed in dH2O, and washed in PBS for 5 min. Immunohistochemisty was performed using the Vectastain Elite ABC-HRP Kit (Rabbit IgG) (PK-6101) using a 1:300 dilution of Optineurin C-TERM rabbit polyclonal antibody (Cayman). Detection was achieved by incubating slides in ImmPACT DAB (SK- 4105). Counterstaining was performed by incubating slides in a 50% hematoxylin solution for 30 s. Slides were then dehydrated with ethanol and mounted with Permount.

### Flow cytometry

Cells were removed from culture plate using HANK’s dissociation buffer. Cells were washed in PBS with 5% FBS then fixed with 4% paraformaldehyde. Cell were washed again in PBS 5% FBS then resuspended in the same buffer for flow cytometry on an Accuri C6 flow cytometer (BD Sciences). Data collection was performed using the BD Accuri C6 Plus v1.0 software. Histograms were prepared in FlowJo v10.0.7 software. For brainstem analysis, tissue was dissociated in 100 µL of 5 mg/mL collagenase IV (Sigma Aldrich) in OptiMem (Gibco). The tissue was spun down at 500 × *g* for 1 min and resuspended in FACS buffer (PBS, 1% BSA). The cell suspension was filtered using 100 µm sterile filter, then blocked using TruStain FcX (BioLegend) according to manufacturer’s protocol. After 2 rounds of centrifugations and washes in 1 mL FACS buffer, the samples were stained for CD3, CD4, and CD8 using 1 µL of antibody and 100 µL (10%) of the cell suspension for 1 h. After 2 rounds of centrifugations and washes in 1 mL FACS buffer, the samples were resuspended in 1 mL FACS buffer and immediately analyzed on an Accuri C6 flow cytometer (BD Sciences).

### Apoptosis detection by flow cytometry

*Optn*+/+ and *Optn*−/− HeLa cells were infected with HSV-1 at 0.1 MOI and harvested at 24 hpi. FITC Annexin V/Dead Cell Apoptosis Kit (InvitrogenTM) was used to detect apoptosis. As per the manufacturer protocol the sample pellets were washed with cold PBS and then suspended in 1x annexin binding buffer. The suspension was mixed with 5 µL FITC Annexin V and 1 µL of a propidium iodide solution (100 µg/mL) for 15 min in the cold. The mixture was diluted with 1X annexin binding buffer. Further flow cytometry (BD AccuriTM C6 Plus flow cytometer) was used to analyze the staining for Annexin V and propidium iodide. Flow data was analyzed using FlowJo software (Tree Star Inc.).

### Plaque assays

After scraping cells from a culture dish cell pellets were resuspended in 1 mL of Opti-Mem. Cell suspension were sonicated for a 5 s pulse at 25% amplitude then immediately serially diluted in Opti-MEM. For tissue samples, the tissue was place in 1 ml of Opti-MEM and sonicated as described. Dilutions are added to Vero cell monolayers and incubated at 37 °C and 5% CO_2_ for 2 h. Following incubation the virus dilution is aspirated and fresh medium (DMEM, 10% FBS, 1% Penn/Strep, 5% methylcelluose w/v) is added to the culture dish. Cells are returned to incubator (37 °C and 5% CO2) for ~3 days or until plaques can be observed. Monolayers are fixed by adding methanol directly to the medium for 10 min at room temperature and then aspirating the methanol-containing medium. The cells are stained with crystal violet (70% water, 20% ethanol, 10% crystal violet stock [1 g/100 mL crystal violet in 20% ethanol]) for 1 h, then aspirated and dried to visually count plaques under a microscope.

### Immunoprecipitation

Cells were scraped from culture dishes and centrifuged to pellet cells. Cell pellets were lysed on ice for 1 h in immunoprecipitation (IP) buffer (250 mM NaCl, 50 mM Tris, 0.5 mM EDTA, 0.5% NP-40) with protease/phosphatase inhibitor added. For the anti-VP16 immunoprecipitation, 10 mM *N*-Ethylmaleimide was added to the IP Buffer immediately before use. Lysates were centrifuged to remove cell debris and the soluble portion was precleared with Isotype control antibody (Santa Cruz) and protein A/G conjugated beads (Santa Cruz) for 1 h with agitation at 4 °C. Beads were pelleted by centrifugation and lysates were moved to new tubes. The lysates were then incubated with isotype or specific antibody (5 µg per 1.7 mL tube with 1 mL lysate) on ice for 1 h. 20 µL of protein A/G beads were added and samples were agitated at 4 °C overnight. The beads containing the immunoprecipitated proteins were pelleted by centrifugation and the unbound portion decanted. The beads were washed four times in IP buffer prior to processing for immunoblot analysis.

### Immunoblot

Cells were scraped from cell culture dish and cells collected in 1.7 mL tubes were centrifuged. Cell pellets were lysed on ice for 30 min using RIPA buffer (Sigma) with added protease and phosphatase inhibitor unless otherwise stated for immunoprecipitation. Lysates were centrifuged to pellet debris and the soluble fraction was mixed with NuPage LDS Sample Buffer (Life Technologies) and β-mercaptoethanol. Samples were incubated on a heat block at 95 °C for 10 min. Electrophoresis was performed using a NuPage 4–12% gradient polyarcrylamide gel (Life Technologies) with NuPage MOPS running buffer (Life Technologies) at 70 V at room temperature. Proteins were transferred to a PVDF membrane using an iBlot 2 system (Thermo Fisher Scientific). Membranes were blocked for 1 h in blocking buffer (5% milk in tris-buffered saline with 0.1% tween-20 (TBST)) followed by overnight primary incubation (1:1000–1:5000 in blocking buffer) with gentle rocking at 4 °C. Membranes were washed three times in TBST before incubation with secondary anti-rabbit or anti-mouse antibody conjugated to HRP (1:10,000 in blocking buffer) for 2 h at room temperature. ECL Femto Substrate (Thermo Fisher Scientific) was used to develop blots and bands were visualized using a Quant 4000 (General Electric) and densitometry analysis was performed in ImageQuant TL and ImageJ software. GAPDH was used as a reference control for sample loading.

### Entry assay

In all, 2 × 10^4^ cells per well were plated in a 96-well format. Cells were confluent after incubation overnight and were then infected at multiple MOIs with gL86 HSV-1. 6 hpi the medium was aspirated, and the cells were washed with PBS. In all, 100 µL of β-galactosidase substrate (0.5% NP40, 3 mg/mL O-nitro-phenyl-β-d-galactopyranoside [Thermo Fisher Scientific]) was added to each well and plated were incubated at 37 °C for 2 h. Colorimetric reaction signal was measured at 410 nm using a GENESIS Pro Plate reader. Alternatively, cells were plated in a 12-well format and infected with gL86 HSV-1 at the same MOIs for the same amount of time. X-gal was used to develop the reaction and the plate was scanned to compare the color visually.

### Infections

Infections were performed by suspending HSV-1 from a thawed stock in Opti-MEM and adding this mixture to cell cultures. Cells are incubated with the virus for 2 h before medium is removed and virus-free growth medium is added to cells. In total, 17 strain was used for in vitro experiments unless otherwise specified.

### Transfection

HeLa cells were plated to be 60–70% confluent in six-well format. In all, 2 µL Lipofectamine 2000 (Thermo Fisher Scientific) was used to transfected 2 µg of plasmid as directed by the manufacturer’s protocol in Opti-MEM. In total, 6 h after transfection cells were washed in growth medium and then incubated for 18 h (24 h total). Cells were transferred to imaging dishes or lysates were collected for experimental use.

### Image analysis

Image analysis was performed in ImageJ software. For time-lapse imaging ImageJ was used to set a color threshold for intense green fluorescence. This threshold allowed the selection and measurement of the infected area relative to total area for each frame, and this data was subsequently graphed in GraphPad Prism. The infection velocity was derived from the change in the infected area between two adjacent time-points, and graphed.

Particle counts were collected in ImageJ by setting color thresholds for each particle type, then using the particle analysis feature in ImageJ to count the number of particles present in the frame.

Colocalization was analyzed in ImageJ. Background fluorescence was subtracted from the raw data of each individual channel image using a rolling ball method with a radius of 30 pixels. Following subtraction, colocalization was quantified by finding the Mander’s correlation coefficient (MCC) using the Coloc 2 plugin. The region of interest used was hand-drawn around the cell or the nucleus to determine the MCC for the nucleus or extranuclear region.

Localization of OPTN was analyzed in ImageJ by similarly subtracting background and drawing regions of interest in the raw data images. The selected regions were measured for signal area and total area and these measurements were used to determine the signal density for the nucleus or extranuclear region.

### Cytokine profiling

Brainstem or draining lymph node samples were collected from mock or 8 dpi mice and profiled using the MILLIPLEX MAP Mouse Cytokine/Chemokine Magnetic Bead Panel - Immunology Multiplex Assay (MCYTOMAG-70K, Millipore Sigma), with the aid of a Millipore Sigma field application scientist. The measurements were analyzed using a K-means clustering algorithm and plotted as heatmaps using the MIT Broad Institute’s Morpheus online tool (https://software.broadinstitute.org/morpheus/).

### Statistics and reproducibility

All statistical analysis and graph making was carried out in GraphPad Prism software, except for flow cytometry histograms and dot plots produced in FlowJo software. Error bars represent ± SEM of at least three independent measurements (*n* = 3). Asterisks denote a significant difference, as determined by two-tailed unpaired Student’s *t* test, Mann–Whitney *U* test, or Logrank test: ^∗^*p* < 0.05; ^∗∗^*p* < 0.01; ^∗∗∗^*p* < 0.001; ns not significant, or two-way ANOVA; ^∗∗∗∗^*p* < 0.0001. All experiments were repeated independently a minimum of three times.

### Ethical approval

The authors have complied with all regulations regarding the use of research animals and the study protocol was approved by the University of Illinois at Chicago Animal Care Committee (protocol: 20-065).

### Reporting summary

Further information on research design is available in the Nature Research Reporting Summary linked to this article.

## Supplementary information

Supplementary Infomation

Reporting Summary

## Data Availability

All data required to understand the study has been made available in the material presented. Data and materials used in this study can be made available through contact with the corresponding author. Source data are provided with this paper.

## Source data

Source Data
