# Supplementary material for: OPTN is a host intrinsic restriction factor against neuroinvasive HSV-1 infection
Source: Nat Commun. 2021 Sep 13;12:5401. doi: 10.1038/s41467-021-25642-z (PMC8437952; doi:10.1038/s41467-021-25642-z)
Supplement: Supplementary file 1 — Supplementary Infomation [file 41467_2021_25642_MOESM1_ESM.pdf]

Supplementary Information

OPTN is a host intrinsic restriction factor against neuroinvasive HSV-1 infection

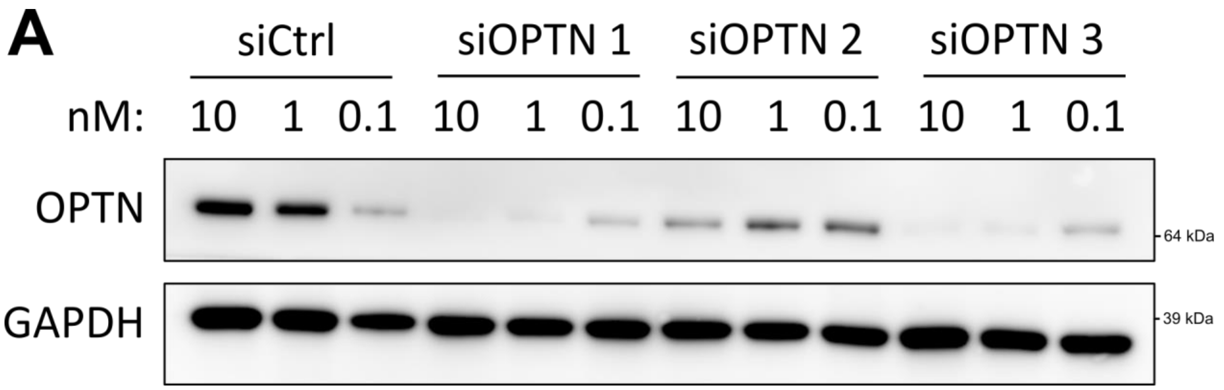

Supplementary Figure 1: siRNA knockdown of OPTN.

(A) Immunoblot against OPTN and reference gene, GAPDH, showing knockdown of OPTN in HCE cells 48 h after transfection. Source data are provided as a Source Data file.

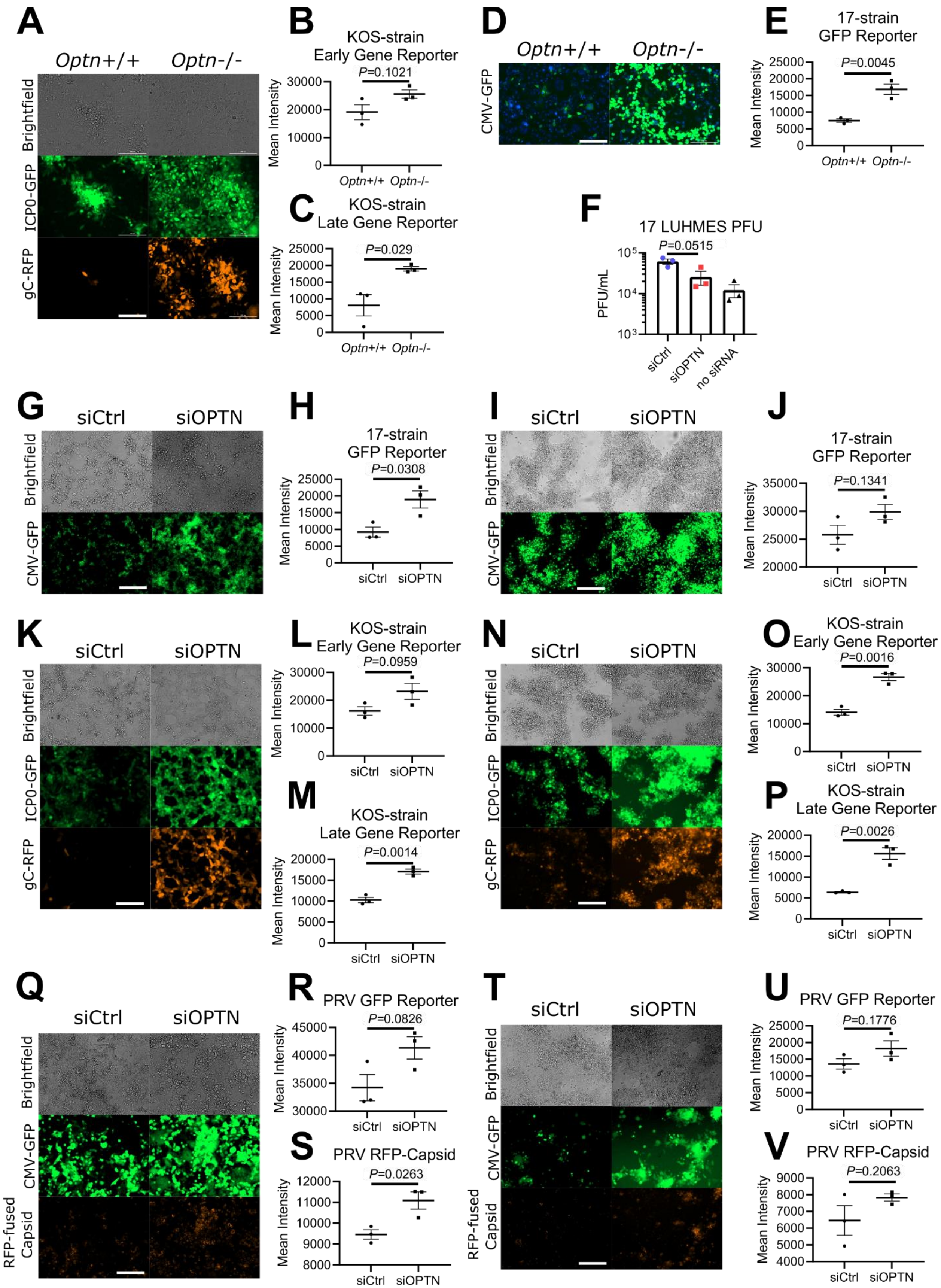
A
Optn+/+
Optn-/-
Brightfield
ICP0-GFP
gC-RFP
B
KOS-strain
Early Gene Reporter
P=0.1021
Mean Intensity
Optn+/+
Optn-/-
C
KOS-strain
Late Gene Reporter
P=0.029
D
CMV-GFP
E
17-strain
GFP Reporter
P=0.0045
F
17 LUHMES PFU
P=0.0515
PFU/mL
siCtrl
siOPTN
no siRNA
G
H
17-strain
GFP Reporter
P=0.0308
I
J
P=0.1341
K
L
KOS-strain
Early Gene Reporter
P=0.0959
M
KOS-strain
Late Gene Reporter
P=0.0014
N
O
KOS-strain
Early Gene Reporter
P=0.0016
P
KOS-strain
Late Gene Reporter
P=0.0026
Q
RFP-fused Capsid
R
PRV GFP Reporter
P=0.0826
S
PRV RFP-Capsid
P=0.0263
T
U
PRV GFP Reporter
P=0.1776
V
PRV RFP-Capsid
P=0.2063

Supplementary Figure 2: OPTN restricts viral infection in strain and cell type dependent manner.

MEF cells derived from *Optn*+/+ or *Optn*-/- mice were infected at 0.1 MOI with either a dual reporter expressing HSV-1 KOS strain or a GFP expressing HSV-1 17-strain for 24 h. (A) Shown for KOS strain infected MEF cells are representative images, (B) GFP fluorescence intensity quantification, and (C) RFP fluorescence intensity. (D) Shown for 17-strain infected MEF cells are representative images and (E) GFP fluorescence intensity. siOPTN or siCtrl transfected HCE or LUHMES cells were infected at 0.1 MOI or 1 MOI respectively with a GFP expressing HSV-1 17-strain for 24 h. (F) Shown are HSV-1 titers from 17-strain infected LUHMES cells 24 hpi. Shown for HCE cells are (G) representative images and (H) fluorescence intensity quantification and shown for LUHMES cells are (I) representative images and (J) fluorescence intensity quantification. siOPTN or siCtrl transfected HCE or LUHMES cells were infected at 0.1 MOI or 1 MOI respectively with a dual reporter expressing HSV-1 KOS strain for 24 h. GFP expression is driven by an early genes promoter and RFP expression is driven by a late gene reporter. (K) Shown for HCE cells are representative images, (L) GFP fluorescence intensity quantification and (M) RFP fluorescence intensity. Shown for LUHMES cells are (N) representative images, (O) GFP fluorescence intensity quantification and (P) RFP fluorescence intensity. siOPTN or siCtrl transfected HCE or LUHMES cells were infected at 0.1 MOI or 1 MOI respectively with a PRV strain expressing GFP from a CMV promoter, and RFP is fused to the viral capsid for 24 h. Shown for HCE cells are (Q) representative images, (R) GFP fluorescence intensity quantification and (S) RFP fluorescence intensity. Shown for LUHMES cells are (T) representative images, (U) GFP fluorescence intensity quantification and (V) RFP fluorescence intensity. All scale bars are 200 µm. All groups represent three biological replicates (n=3). Data are presented as mean values +/- SEM (B-C, E-F, H, J, L-M, O-P, R-S, U-V). Two-tailed Student's t-test was performed for statistical analysis ($\alpha = 0.05$). $*p < 0.05$; $**p < 0.01$; ns, not significant. Source data are provided as a Source Data file.

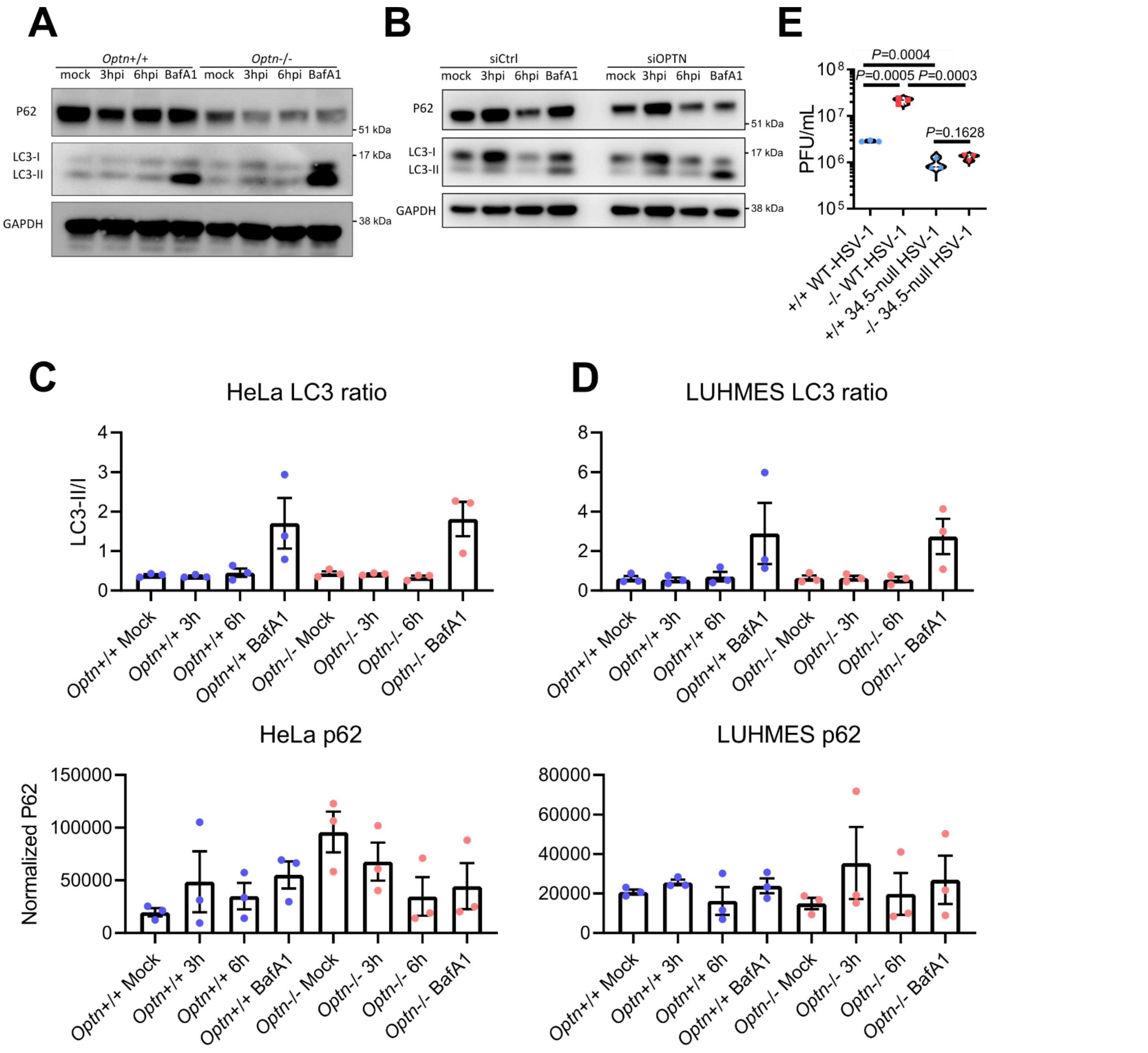

Supplementary Figure 3: Total autophagy levels are not changed by OPTN deficiency.

(A) Immunoblot of *Optn*+/+ and -/- cells infected with HSV-1 at 1 MOI. Blots were against autophagy markers P62 and LC3, and OPTN and the internal reference gene GAPDH. (B) Immunoblot of siOPTN or siCtrl transfected LUHMES cells infected with HSV-1 at 2.5 MOI. Blots were against autophagy markers P62 and LC3, and OPTN and the internal reference gene GAPDH. (C) Quantitation of HeLa lysate band densities to determine LC3-II/I ratio and normalized p62 levels. (D) Quantitation of LUHMES lyate band densities to determine LC3-II/I ratio and normalized p62 levels. (E) Plaque forming units (PFU) of *Optn*+/+ and -/- cells infected with 17-strain wildtype or ICP34.5 null 17-strain HSV-1 at 0.1 MOI for 24 h. n=3 replicates for all groups. Data are presented as mean values +/- SEM (C-D). Two-tailed Student's t-test was performed for statistical analysis ($\alpha = 0.05$). $***p < 0.001$. Source data are provided as a Source Data file.

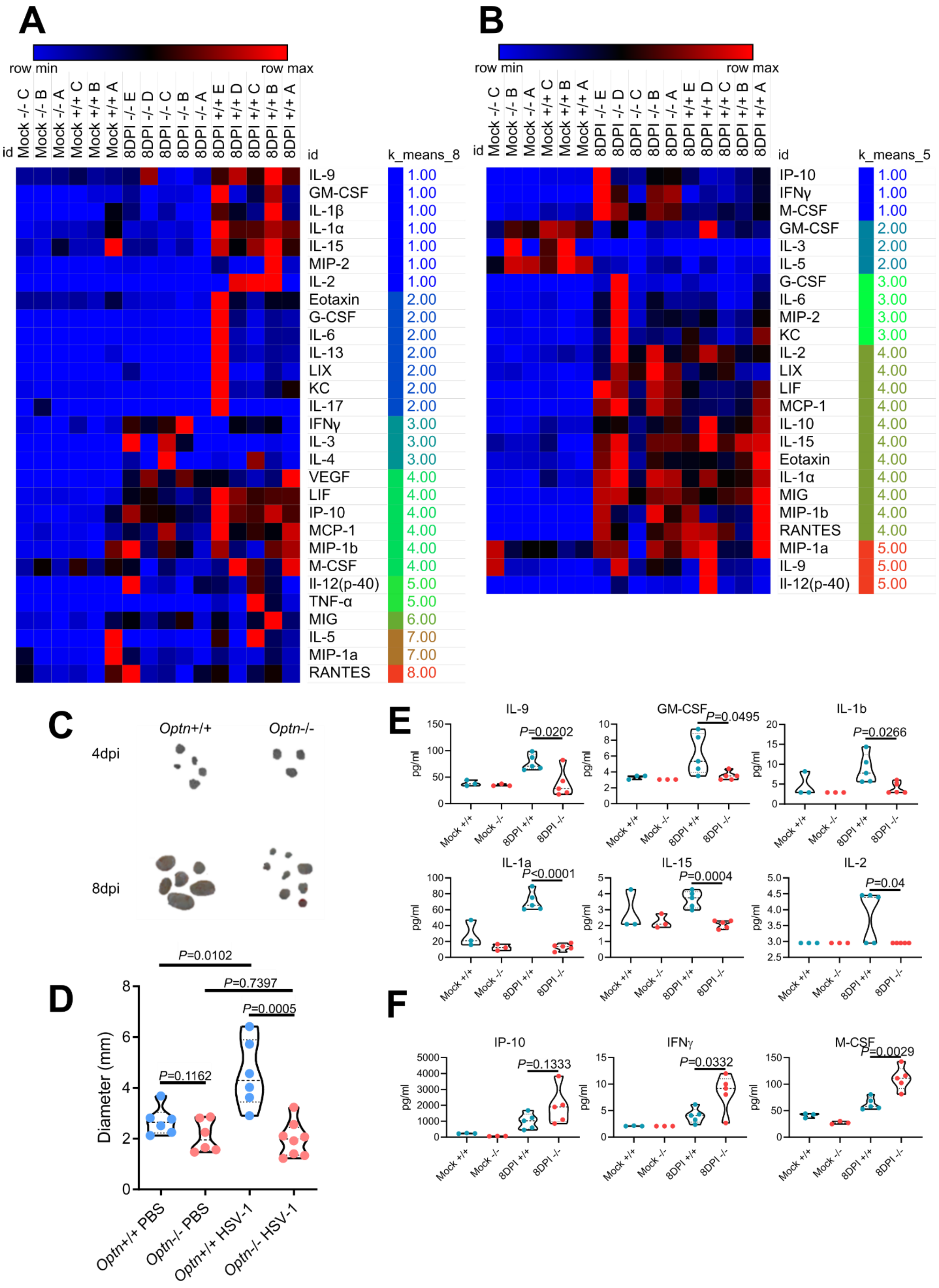
A
row min
row max
id
Mock -/- C
Mock -/- B
Mock -/- A
Mock +/+ C
Mock +/+ B
Mock +/+ A
8DPI -/- E
8DPI -/- D
8DPI -/- C
8DPI -/- B
8DPI -/- A
8DPI +/+ E
8DPI +/+ D
8DPI +/+ C
8DPI +/+ B
8DPI +/+ A
id
k_means_8
IL-9 1.00
GM-CSF 1.00
IL-1β 1.00
IL-1α 1.00
IL-15 1.00
MIP-2 1.00
IL-2 1.00
Eotaxin 2.00
G-CSF 2.00
IL-6 2.00
IL-13 2.00
LIX 2.00
KC 2.00
IL-17 2.00
IFNγ 3.00
IL-3 3.00
IL-4 3.00
VEGF 4.00
LIF 4.00
IP-10 4.00
MCP-1 4.00
MIP-1b 4.00
M-CSF 4.00
Il-12(p-40) 5.00
TNF-α 5.00
MIG 6.00
IL-5 7.00
MIP-1a 7.00
RANTES 8.00
B
row min
row max
id
Mock -/- C
Mock -/- B
Mock -/- A
Mock +/+ C
Mock +/+ B
Mock +/+ A
8DPI -/- E
8DPI -/- D
8DPI -/- C
8DPI -/- B
8DPI -/- A
8DPI +/+ E
8DPI +/+ D
8DPI +/+ C
8DPI +/+ B
8DPI +/+ A
id
k_means_5
IP-10 1.00
IFNγ 1.00
M-CSF 1.00
GM-CSF 2.00
IL-3 2.00
IL-5 2.00
G-CSF 3.00
IL-6 3.00
MIP-2 3.00
KC 3.00
IL-2 4.00
LIX 4.00
LIF 4.00
MCP-1 4.00
IL-10 4.00
IL-15 4.00
Eotaxin 4.00
IL-1α 4.00
MIG 4.00
MIP-1b 4.00
RANTES 4.00
MIP-1a 5.00
IL-9 5.00
Il-12(p-40) 5.00
C
Optn+/+
Optn-/-
4dpi
8dpi
D
P=0.0102
P=0.7397
P=0.0005
P=0.1162
Diameter (mm)
Optn+/+ PBS
Optn-/- PBS
Optn+/+ HSV-1
Optn-/- HSV-1
E
IL-9
P=0.0202
GM-CSF
P=0.0495
IL-1b
P=0.0266
IL-1a
P<0.0001
IL-15
P=0.0004
IL-2
P=0.04
pg/ml
Mock +/+
Mock -/-
8DPI +/+
8DPI -/-
F
IP-10
P=0.1333
IFNγ
P=0.0332
M-CSF
P=0.0029
pg/ml
Mock +/+
Mock -/-
8DPI +/+
8DPI -/-

Supplementary Figure 4: OPTN deficiency alters host cytokine expression.

(A) Heat map of cytokine protein levels in *Optn*+/+ or -/- mouse dLN lysates. K-means cluster group indicated right of rows. Each column represents the cytokine profile of an individual mouse dLN. (B) Heatmap of cytokine protein levels in *Optn*+/+ or -/- mouse brainstem lysates 8 dpi. K-means group indicated right of rows. Each column represents the cytokine profile of an individual mouse brainstem. (C) Image of representative dLNs. (D) Quantification of dLN diameters for *Optn* +/+ or -/- mice 8 dpi. (*Optn* +/+ PBS, *Optn* -/- PBS, *Optn* +/+ HSV-1: n = 5 lymph nodes; *Optn* -/- HSV-1: n = 7 lymph nodes) (E) Quantitation of tissue protein levels of cytokines present in k-means cluster 1 from mouse dLNs. Mock groups: n = 3 mice, HSV-1 8 dpi groups: n = 5 mice. (F) Quantitation of tissue protein levels of cytokines present in k-means cluster 1 from mouse brainstems. Mock groups: n = 3 mice, HSV-1 8 dpi groups: n = 5 mice. Two-tailed Student's t-test was performed for statistical analysis ($\alpha = 0.05$). $*p < 0.05$; $**p < 0.01$; $***p < 0.001$, $****p < 0.0001$, ns, not significant. Source data are provided as a Source Data file.

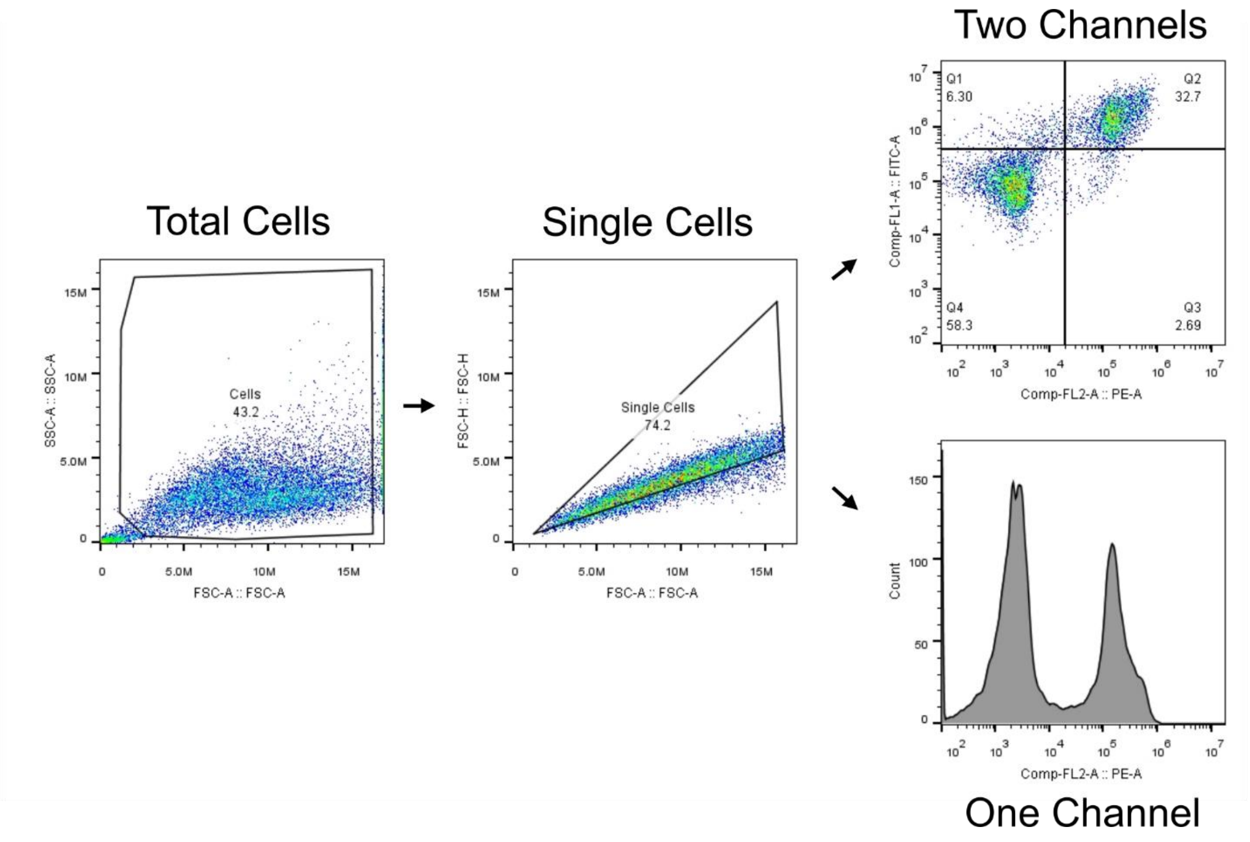

Flow cytometry gating: Simple gating strategy was used to gate all cells (left) based on SSC and FSC areas, then single cells (middle) based on FSC height and FSC area, then single cells were analyzed for a one- or two-color stain experiment as depicted in the example plots (right).

Primer name	Primer sequence
viral glycoprotein gD forward primer	5′-TACAACCTGACCATCGCTTC-3′
viral glycoprotein gD reverse primer	5′-GCCCCCAGAGACTTGTTGTA-3′
GAPDH forward primer	5′-TCCACTGGCGTCTTCACC-3′
GAPDH reverse primer	5′-GGCAGAGATGATGACCCTTTT-3′

Supplementary Table 1: Primer sequences
