# Supplementary material for: OPTN is a host intrinsic restriction factor against neuroinvasive HSV-1 infection
Source: Nat Commun. 2021 Sep 13;12:5401. doi: 10.1038/s41467-021-25642-z (PMC8437952; doi:10.1038/s41467-021-25642-z)
Supplement: Supplementary file 2 — Reporting Summary [file 41467_2021_25642_MOESM2_ESM.pdf]

nature research

Corresponding author(s): Dr. Deepak Shukla

Last updated by author(s): Jul 12, 2021

Reporting Summary

Nature Research wishes to improve the reproducibility of the work that we publish. This form provides structure for consistency and transparency in reporting. For further information on Nature Research policies, see our Editorial Policies and the Editorial Policy Checklist.

Statistics

For all statistical analyses, confirm that the following items are present in the figure legend, table legend, main text, or Methods section.

n/a	Confirmed	
☐	☒	The exact sample size (n) for each experimental group/condition, given as a discrete number and unit of measurement
☐	☒	A statement on whether measurements were taken from distinct samples or whether the same sample was measured repeatedly
☐	☒	The statistical test(s) used AND whether they are one- or two-sided<br>*Only common tests should be described solely by name; describe more complex techniques in the Methods section.*
☐	☒	A description of all covariates tested
☐	☒	A description of any assumptions or corrections, such as tests of normality and adjustment for multiple comparisons
☐	☒	A full description of the statistical parameters including central tendency (e.g. means) or other basic estimates (e.g. regression coefficient) AND variation (e.g. standard deviation) or associated estimates of uncertainty (e.g. confidence intervals)
☐	☒	For null hypothesis testing, the test statistic (e.g. F, t, r) with confidence intervals, effect sizes, degrees of freedom and P value noted<br>*Give P values as exact values whenever suitable.*
☒	☐	For Bayesian analysis, information on the choice of priors and Markov chain Monte Carlo settings
☐	☒	For hierarchical and complex designs, identification of the appropriate level for tests and full reporting of outcomes
☒	☐	Estimates of effect sizes (e.g. Cohen's d, Pearson's r), indicating how they were calculated

Our web collection on statistics for biologists contains articles on many of the points above.

Software and code

Policy information about availability of computer code

Data collection	BD Accuri C6 Plus v1.0, ZEN Black, and Biotek GEN5 v3.04 Imager softwares were used for data collection in this study.
Data analysis	Fiji ImageJ 1.52p, Graph Pad Prism 8, FlowJo v10.0.7, and MIT Broad Institute's Morpheus online tool (https://software.broadinstitute.org/morpheus/) were used for data analysis in this study.

For manuscripts utilizing custom algorithms or software that are central to the research but not yet described in published literature, software must be made available to editors and reviewers. We strongly encourage code deposition in a community repository (e.g. GitHub). See the Nature Research guidelines for submitting code & software for further information.

Data

Policy information about availability of data

All manuscripts must include a data availability statement. This statement should provide the following information, where applicable:

- Accession codes, unique identifiers, or web links for publicly available datasets
- A list of figures that have associated raw data
- A description of any restrictions on data availability

All data required to understand the study has been made available in the material presented. Data and materials used in this study can be made available through contact with the corresponding author.

Field-specific reporting

Please select the one below that is the best fit for your research. If you are not sure, read the appropriate sections before making your selection.

☒ Life sciences ☐ Behavioural & social sciences ☐ Ecological, evolutionary & environmental sciences

For a reference copy of the document with all sections, see nature.com/documents/nr-reporting-summary-flat.pdf

Life sciences study design

All studies must disclose on these points even when the disclosure is negative.

Sample size	Sample sizes were based on institutionally approved protocols for set numbers of genetically identical mice ranging from 4-10 (one or two cages depending on availability from breeding colony and age). Minimum of n=3 was used for all animal experiments and the total number of changed with the availability of animals from the breeding colony. The sample size of each experiment was determined based on similar experiments previously conducted by our group. Additionally, a retrospective power analysis based on 5x10^5 PFU HSV-1 McKrae ocularly infected OPTN-WT or OPTN-KO mice brainstem HSV-1 tissue titers reveals that 4 mice per infected group is required to generate relevant mean results with significance or α at 0.05 level and power level at 80%. Calculations were performed using powerandsamplesize.com (HyLown Consulting LLC) in accordance with reference: Chow S, Shao J, Wang H. 2008. Sample Size Calculations in Clinical Research. 2nd Ed. Chapman & Hall/CRC Biostatistics Series. page 58.
Data exclusions	No data were excluded from study
Replication	Multiple types of experiments and multiple models were used to test the hypotheses addressed in this study. Multiple biological replicates for each experiment were conducted to reproduce results. The experiments were replicated a minimum of 3 times to ensure consistent results. Results were consistently reproducible.
Randomization	Randomization was not used in this study. The mouse model used compared two genotypes, and mice were grouped by genetic strain. Experiments not using mice were performed in cell culture dishes containing multiple wells. Different treatments for an experiment were all included in a single plate for a replicate to control for confounding factors due to culturing and handling.
Blinding	Blinding was not relevant to this study as there was minimal scoring, and most measures were quantifiable by standard cellular or biochemical assays. All the results were objective and none were subjective to interpretation and hence blinding was unnecessary. Additionally there was no clinical enrollment of participants that would require blinding.

Reporting for specific materials, systems and methods

We require information from authors about some types of materials, experimental systems and methods used in many studies. Here, indicate whether each material, system or method listed is relevant to your study. If you are not sure if a list item applies to your research, read the appropriate section before selecting a response.

Materials & experimental systems

n/a	Involved in the study	
☐	☒	Antibodies
☐	☒	Eukaryotic cell lines
☒	☐	Palaeontology and archaeology
☐	☒	Animals and other organisms
☐	☒	Human research participants
☒	☐	Clinical data
☒	☐	Dual use research of concern

Methods

n/a	Involved in the study	
☒	☐	ChIP-seq
☐	☒	Flow cytometry
☒	☐	MRI-based neuroimaging

Antibodies

Antibodies used

The following antibodies and stains were used in this study for imaging:
DAPI (D9542, Sigma),
NucBlue™ Live ReadyProbes™ Reagent (Thermo Fisher, R37605),
Mouse monoclonal to LAMP1 (Abcam, ab25630, [H4A3]),
Rabbit polyclonal anti-LC3B (Novus, NB100-2220),
Mouse monoclonal anti-HSV1 + HSV2 VP16 (Abcam, ab110226, [LP1]),
Goat anti-Mouse IgG (H+L) Highly Cross-Adsorbed Secondary Antibody, Alexa Fluor 546 (Thermo Fisher, A-11030),
Goat anti-Rabbit IgG (H+L) Highly Cross-Adsorbed Secondary Antibody, Alexa Fluor 647 (Thermo Fisher, A-21245),
Rabbit polyclonal anti- Optineurin (CTerm) (Cayman Chemical, No. 100000),
and anti-mouse CD8a APC-conjugated monoclonal antibody (Tonbo biosciences, 20-1886-U100, [clone 2.43]).
The following antibodies were used for immunoblot:

Mouse monoclonal anti-GAPDH (Santa Cruz, sc-69778, [7B]),
Goat anti-Mouse IgG (H+L) Highly Cross-Adsorbed Secondary Antibody HRP (Thermo Fisher, 31432),
Goat anti-Rabbit IgG (H+L) Cross-Adsorbed Secondary Antibody HRP (Thermo Fisher, G-21234),
Mouse monoclonal anti-FLAG (Sigma, F1804, [M2]),
Mouse monoclonal anti-HSV1 ICP0 (Abcam, ab6513, [5H7]),
Rabbit monoclonal anti-p-S177 OPTN (Cell Signaling Technologies, 57548S),
Mouse monoclonal anti-HSV1 + HSV2 VP16 (Abcam, ab110226, [LP1]),
Rabbit polyclonal anti- Optineurin (CTerm) (Cayman Chemical, No. 100000).
The following antibodies were used for immunoprecipitation:
Mouse monoclonal anti-FLAG (Sigma, F1804, [M2]),
normal mouse IgG (Santa Cruz, sc-2025),
and additionally, Protein A/G PLUSAgarose beads (Santa Cruz, sc-2003) were used.
For flow cytometry the following antibodies were used:
and anti-mouse CD8a APC-conjugated monoclonal antibody (Tonbo biosciences, 20-1886-U100, [clone 2.43]).
anti-mouse CD4 PE-conjugated monoclonal antibody (Tonbo biosciences, 50-0042-U100, [RM 4-5]).

Validation

Mouse monoclonal [H4A3] to LAMP1 (Abcam): "Our Abpromise guarantee covers the use of ab25630 in the following tested applications." Reacts with: Human. Suitable for: ICC/IF, Flow Cyt, WB, IHC-P. 157 References:
Taverner A et al. Cholix protein domain I functions as a carrier element for efficient apical to basal epithelial transcytosis. Tissue Barriers 8:1710429 (2020).
Kang L et al. The mitochondria-targeted anti-oxidant MitoQ protects against intervertebral disc degeneration by ameliorating mitochondrial dysfunction and redox imbalance. Cell Prolif 53:e12779 (2020).
Larios J et al. ALIX- and ESCRT-III-dependent sorting of tetraspanins to exosomes. J Cell Biol 219:N/A (2020).

Rabbit polyclonal anti-LC3B (Novus): Novus validated by genetic and biological strategies. Reactivity: Hu, Mu, Rt, Po, Av, Ba, Bv, Ca, Ch, Gp, Ha, In, Pm, Pm, Rb, SyHa, Ze. Suitable for WB, Simple Western, ELISA, Flow, IB, ICC/IF, IHC, IHC-Fr, IHC-P, IP, PLA, ChIP, KD, KO. 1063 references:
1. Yu, L., Chen, Y., & Tooze, S. A. (2018). Autophagy pathway: Cellular and molecular mechanisms. Autophagy. https://doi.org/10.1080/15548627.2017.1378838
2. Forrester, A., De Leonibus, C., Grumati, P., Fasana, E., Piemontese, M., Staiano, L.,... Settembre, C. (2019). A selective ER -phagy exerts procollagen quality control via a Calnexin- FAM 134B complex. The EMBO Journal. https://doi.org/10.15252/embj.201899847
3. He, X., Zhu, Y., Zhang, Y., Geng, Y., Gong, J., Geng, J.,... Zhong, H. (2019). RNF34 functions in immunity and selective mitophagy by targeting MAVS for autophagic degradation. The EMBO Journal. https://doi.org/10.15252/embj.2018100978

Mouse monoclonal [LP1] anti-HSV1 + HSV2 VP16 (Abcam): "Our Abpromise guarantee covers the use of ab110226 in the following tested applications."
Specificity: This antibody is specific for Herpes simplex virus tegument protein VP16. This antibody will cross react with HSV1 and HSV2. Suitable for: ICC/IF, IP, WB. 17 references:
Soh TK et al. Temporal Proteomic Analysis of Herpes Simplex Virus 1 Infection Reveals Cell-Surface Remodeling via pUL56-Mediated GOPC Degradation. Cell Rep 33:108235 (2020).
Harrison KS et al. Antagonizing the Glucocorticoid Receptor Impairs Explant-Induced Reactivation in Mice Latently Infected with Herpes Simplex Virus 1. J Virol 93:N/A (2019).
Zhu L & Jones C The canonical Wnt/ß-catenin signaling pathway stimulates herpes simplex virus 1 productive infection. Virus Res 256:29-37 (2018).

Rabbit polyclonal anti- Optineurin (C-Term) (Cayman Chemical): Company validated for Western Blot and IHC. This study shows genetic validation of specificity through knockout cell line. Applications: IHC and WB. Species: Human.
Product Citations
Falcon, B., Noad, J., McMahon, H., et al. Galectin-8-mediated selective autophagy protects against seeded tau aggregation. J. Biol. Chem. 293(7), 2438-2451 (2018).
Silva, I.A.L., Conceiçao, I., Gagnon, E., et al. Molecular effect of an OPTN common variant associated to Paget's disease of bone. PLoS One 13(5), e0197543 (2018).
Paulus, J.D., and Link, B.A. Loss of optineurin in vivo results in elevated cell death and alters axonal trafficking dynamics. PLoS One 9 (10), e109922 (2014).
Newman, A.C., Scholefield, C.L., Kemp, A.J., et al. TBK1 kinase addiction in lung cancer cells is mediated via autophagy of Tax1bp1/Ndp52 and non-canonical NF-kB signalling. PLoS One 7(11), e50672 (2012).
Journo, C., Filipe, J., About, F., et al. NRP/optineurin cooperates with TAX1BP1 to potentiate the activation of NF-kappaB by human T-lymphotropic virus Type 1 tax protein. PLoS Pathog. 5(7), e1000521 (2009).
anti-mouse CD8a APC-conjugated monoclonal [clone 243] antibody (Tonbo biosciences): "Tonbo Biosciences tests all antibodies by flow cytometry."

Mouse monoclonal [7B] anti-GAPDH (Santa Cruz): Company provides data for immunoblot usage. Recommended for detection of GAPDH of human origin by WB, IP and ELISA. 73 references:
PMID: # 33972507 Garcia-Contreras, M., Thakor, AS., et al. 2021. Cell Death Discov. 7: 98.
PMID: # 30628702 2019. Mol Med Rep. 19: 1883-1890.
PMID: # 31432121 2019. Mol Med Rep. 20: 3175-3181.
PMID: # 30260431 Avolio, R. et al. 2018. Nucleic Acids Res.
PMID: # 29241478 Lv, L. et al. 2018. Oncol. Res. 26: 775-783.

Mouse monoclonal [M2] anti-FLAG (Sigma): species reactivity: all. For highly sensitive and specific detection of FLAG fusion proteins

by immunoblotting, immunoprecipitation (IP) , immunohistochemisty, immunofluorescence and immunocyotchemistry. 4838 references:
Monika Srivastava et al. Nature communications, 6, 6253-6253 (2015-02-24)
Yalan Liu. Virology, 154-154 (2016)

Mouse monoclonal [5H7] anti-HSV1 ICP0 (Abcam): Species: Herpes virus. Suitable for: WB, ELISA, ICC/IF. "Our Abpromise guarantee covers the use of ab6513 in the following tested applications." 27 references:
Furey C et al. TACC3 Regulates Microtubule Plus-End Dynamics and Cargo Transport in Interphase Cells. Cell Rep 30:269-283.e6 (2020).
Subramanian G et al. The interferon-inducible protein TDRD7 inhibits AMP-activated protein kinase and thereby restricts autophagy-independent virus replication. J Biol Chem 295:6811-6822 (2020).
Glanz A et al. High Throughput Screening of FDA-Approved Drug Library Reveals the Compounds that Promote IRF3-Mediated Pro-Apoptotic Pathway Inhibit Virus Replication. Viruses 12:N/A (2020).

Rabbit monoclonal anti-p-S177 OPTN (Cell Signaling Technologies): "As a company of scientists, we understand how important it is to work with antibodies that are specific and consistent from lot-to-lot. To ensure product performance, we validate all of our antibodies, in-house, in multiple research applications. If a product does not perform in your experiment as described on our website or datasheet, please contact your local CST office or nearest distributor (see below for contact information) within 12 months of product receipt. The same expert scientists who produced your antibody will guide you through a few simple troubleshooting steps, and if your issue is not resolved, we will replace the antibody at no cost to you, or provide you with a credit." Suitable for Western blot and Immunoprecipitation. Data provided by company on website regarding usage.

anti-mouse CD4 PE-conjugated monoclonal [clone RM 4-5] antibody (Tonbo biosciences): "Tonbo Biosciences tests all antibodies by flow cytometry." Application: Flow cytometry, Immunohistochemistry, Immunofluorescence microscopy. Species: Mouse. 9 references:
Willinger T and Flavell RA. 2012. Proc. Natl. Acad. Sci. 109:8670-8675. (Flow cytometry)
Stephen TL, Wilson BS, and Laufer TM. 2012. Proc. Natl. Acad. Sci. 109: 7415-7420. (Immunofluorescence microscopy)
Poitrasson-Riviere M, Bienvenu B, Le Campion A, Becourt C, Martin B, and Lucas B. 2008. J. Immunol. 180:7294-7304. (Immunohistochemistry – paraffin embedded tissue)
Sorg H, Lorch B, Jaster R, Fitzner B, Ibrahim S, Holzhueter S, Nizze H, and Vollmar B. 2008. Am. J. Physio. Gastrointest. Liver Physiol. 295: G1274-1280. (Immunohistochemistry - paraffin embedded tissue)
Menke J, Lucas JA, Zeller GC, Keir ME, Huang XR, Tsuboi N, Mayadas TN, Lan HY, Sharpe AH, and Kelley VR. 2007. J. Immunol. 179: 7466-7477. (Immunohistochemistry – frozen tissue)
Irie J, Wu Y, Wicker LS, Rainbow D, Nalesnik MA, Hirsch R, Peterson LB, Leung PS, Cheng C, Mackay IR, Gershwin ME, and Ridgway WM. 2006. J Exp Med. 203(5):1209-19. (Immunohistochemistry – frozen tissue)
Bosselut R, Zhang W, Ashe JM, Kopacz JL, Samelson LE, and Singer A. 1999. J. Exp. Med. 190: 1517-1526. (Immunoprecipitation)
Shi Y, Kaliyaperumal A, Lu L, Southwood S, Sette A, Michaels MA, and Datta SK. 1998. J. Exp. Med. 187:367-378. (Blocking)
Whiteland JL, Nicholls SM, Shimeld C, Easty DL, Williams NA, and Hill TJ. 1995. J. Histochem. Cytochem. 43:313-320. (Immunohistochemistry – frozen tissue, zinc-fixed paraffin embedded tissue)

and anti-mouse CD8a APC-conjugated monoclonal antibody (Tonbo biosciences, 20-1886-U100, [clone 2.43]). "Tonbo Biosciences tests all antibodies by flow cytometry." Application: Flow cytometry, Immunohistochemistry – OCT embedded frozen tissue, in vitro activation. Species: Mouse. References:
Lin J-S, Szaba FM, Kummer LW, Chromy BA, and Smiley ST. 2011. J. Immunol. 187: 897-904. (in vivo depletion)
Wozniak KL, Young ML, and Wormley FL. 2011. Clin. Vaccine Immunol. 18(5):717-723. (in vivo depletion)
Hufford MM, Kim TS, Sun J, and Braciale TJ. 2011. J. Exp. Med. 208: 167-180 (in vivo depletion)
Ou R, zhang M, Huang L, Flavell RA, Koni PA, and Moskophidis D. 2008. J. Virol. 82:2952-2965. (Immunohistochemistry – OCT embedded frozen tissue)
Bosselut R, Zhang W, Ashe JM, Kopacz JL, Samelson LE, and Singer A. 1999. J. Exp. Med. 190: 1517-1526. (in vitro activation)
Davies A, Kalb, S, Liang B, Aldrich CJ, Lemonnier FA, Jiang H, Cotter R, and Soloski MJ. 2003. J. Immunol. 170: 5027-5033. (Blocking)

Eukaryotic cell lines

Policy information about cell lines

Cell line source(s)	[HeLa Optn-/- and parental strain were provided by Dr. Richard Youle (National Institutes of Health)] [LUHMES cells (ATCC) were provided by Dr. David Bloom (University of Florida)] [HCE cells were provided by Dr. Kozaburo Hayashi (National Eye Institute, Bethesda, MD)][Vero cells were acquired from ATCC]
Authentication	Cell lines were authenticated using STR analysis
Mycoplasma contamination	Cell lines were not tested for mycoplasma contamination
Commonly misidentified lines (See ICLAC register)	No commonly misidentified lines were used

Animals and other organisms

Policy information about studies involving animals; ARRIVE guidelines recommended for reporting animal research

Laboratory animals	Optn -/- or +/+ mice were generated on a C57/b6j mouse background. Male and female mice were used. 8 - 16 week old mice were

	used.
Wild animals	Study did not contain wild animals.
Field-collected samples	Study did not involve collection of field samples.
Ethics oversight	The mouse work in this studying was approved by the Animal Care Committee (ACC) through the Office of Animal Care and Institutional Biosafety (OACIB) within the Office of the Vice Chancellor for Research for the University of Illinois at Chicago (Protocol Number: 20-065)

Note that full information on the approval of the study protocol must also be provided in the manuscript.

Human research participants

Policy information about studies involving human research participants

Population characteristics	Human research participants:<br>Human nervous system tissues analyzed by immunohistochemistry for optineurin expression were derived from autopsies. Sections of de-identified paraffin-embedded formalin fixed brain tissue from patients with diagnosis of amyotrophic lateral sclerosis (ALS) or without neurological disease were obtained through the University of Illinois Biorepository using Institutional Review Board (IRB)-approved protocols (protocol # 2014-1019). Sections of de-identified paraffin-embedded formalin fixed brain tissue from an autopsy on a patient with the diagnosis of herpes simplex virus encephalitis (HSE) were obtained from Dr. Randall Woltjer of the Alzheimer's Disease Center (ADC) at Oregon Health and Science University (OHSU) using Institutional Review Board (IRB)-approved protocols.<br><br>Population characteristics:<br>Case/diagnosis Age Gender<br>Normal control 75 Female<br>ALS 56 Male<br>HSE 76 Female
Recruitment	Recruitment: Sections of de-identified paraffin-embedded formalin fixed brain tissue from patients with diagnosis of amyotrophic lateral sclerosis (ALS) or without neurological disease were obtained through the University of Illinois Biorepository using Institutional Review Board (IRB)-approved protocols (protocol # 2014-1019). Sections of de-identified paraffin-embedded formalin fixed brain tissue from an autopsy on a patient with the diagnosis of herpes simplex virus encephalitis (HSE) were obtained from Dr. Randall Woltjer of the Alzheimer's Disease Center (ADC) at Oregon Health and Science University (OHSU) using Institutional Review Board (IRB)-approved protocols.
Ethics oversight	Ethics oversight: University of Illinois at Chicago Office for Protection of Research Subjects.<br>"The UIC Office for Protection of Research Subjects received your "Determination of Whether an Activity Represents Human Subjects Research" application, and has determined that this activity does not meet the definition of human subject research as defined by 45 CFR 46.102(f)."

Note that full information on the approval of the study protocol must also be provided in the manuscript.

Flow Cytometry

Plots

Confirm that:

- [x] The axis labels state the marker and fluorochrome used (e.g. CD4-FITC).
- [x] The axis scales are clearly visible. Include numbers along axes only for bottom left plot of group (a 'group' is an analysis of identical markers).
- [x] All plots are contour plots with outliers or pseudocolor plots.
- [x] A numerical value for number of cells or percentage (with statistics) is provided.

Methodology

Sample preparation	Cultured cells were removed from plate using enzyme free, Hank's balanced salt solution cell dissociation buffer before being passed through a 100 micron strainer, then stained with the indicated reagent. For tissue, collagenase in Opti-MEM was used to dissociate tissue into single cells at 37C. Cells were passed through 100 micron filter, stained, then fixed in 4% PFA.
Instrument	BD Accuri C6
Software	BD Accuri C6 Plus v1.0 was used for data collection. FlowJo v10.0.7 was used for analysis
Cell population abundance	No sorting was performed. Only analysis for fluorescent markers was performed.
Gating strategy	A simple gating strategy was used to gate all cells based on SSC and FSC areas, then single cells based on FSC height and FSC

area, then single cells were analyzed for a one or two color stain experiment.

☒ Tick this box to confirm that a figure exemplifying the gating strategy is provided in the Supplementary Information.
